# Cerium(IV) chitosan-based hydrogel composite for efficient adsorptive removal of phosphates(V) from aqueous solutions

**DOI:** 10.1038/s41598-023-40064-1

**Published:** 2023-08-11

**Authors:** Łukasz Wujcicki, Tomasz Mańdok, Wiktoria Budzińska-Lipka, Karolina Pawlusińska, Natalia Szozda, Gabriela Dudek, Krzysztof Piotrowski, Roman Turczyn, Maciej Krzywiecki, Alicja Kazek-Kęsik, Joanna Kluczka

**Affiliations:** 1 Faculty of Chemistry, Silesian University of Technology, Ks. M. Strzody 9, 44-100 Gliwice, Poland; 2 Department of Physical Chemistry and Technology of Polymers, Faculty of Chemistry, Silesian University of Technology, Ks. M. Strzody 9, 44-100 Gliwice, Poland; 3 Department of Chemical Engineering and Process Design, Faculty of Chemistry, Silesian University of Technology, Ks. M. Strzody 7, 44-100 Gliwice, Poland; 4 Institute of Physics - Centre for Science and Education, Silesian University of Technology, Konarskiego 22B, 44-100 Gliwice, Poland; 5 Department of Inorganic, Analytical Chemistry and Electrochemistry, Faculty of Chemistry, Silesian University of Technology, B. Krzywoustego 6, 44-100 Gliwice, Poland; 6 Centre for Organic and Nanohybrid Electronics, Silesian University of Technology, Konarskiego 22B, 44-100 Gliwice, Poland

**Keywords:** Pollution remediation, Biopolymers, Chemical engineering

## Abstract

The excess presence of phosphate(V) ions in the biosphere is one of the most serious problems that negatively affect aqueous biocenosis. Thus, phosphates(V) separation is considered to be important for sustainable development. In the presented study, an original cerium(IV)-modified chitosan-based hydrogel (Ce-CTS) was developed using the chemical co-precipitation method and then used as an adsorbent for efficient removal of phosphate(V) ions from their aqueous solutions. From the scientific point of view, it represents a completely new physicochemical system. It was found that the adsorptive removal of phosphate(V) anions by the Ce-CTS adsorbent exceeded 98% efficiency which is ca. 4-times higher compared with the chitosan-based hydrogel without any modification (non-cross-linked CTS). The best result of the adsorption capacity of phosphates(V) on the Ce-CTS adsorbent, equal to 71.6 mg/g, was a result of adsorption from a solution with an initial phosphate(V) concentration 9.76 mg/dm^3^ and pH 7, an adsorbent dose of 1 g/dm^3^, temperature 20 °C. The equilibrium interphase distribution data for the Ce-CTS adsorbent and aqueous solution of phosphates(V) agreed with the theoretical Redlich-Peterson and Hill adsorption isotherm models. From the kinetic point of view, the pseudo-second-order model explained the phosphates(V) adsorption rate for Ce-CTS adsorbent the best. The specific effect of porous structure of adsorbent influencing the diffusional mass transfer resistances was identified using Weber-Morris kinetic model. The thermodynamic study showed that the process was exothermic and the adsorption ran spontaneously. Modification of CTS with cerium(IV) resulted in the significant enhancement of the chitosan properties towards both physical adsorption (an increase of the point of zero charge of adsorbent), and chemical adsorption (through the presence of Ce(IV) that demonstrates a chemical affinity for phosphate(V) anions). The elaborated and experimentally verified highly effective adsorbent can be successfully applied to uptake phosphates(V) from aqueous systems. The Ce-CTS adsorbent is stable in the conditions of the adsorption process, no changes in the adsorbent structure or leaching of the inorganic filling were observed.

## Introduction

Phosphorus is an element of great biological and industrial importance. It is one of the elements necessary for the proper growth and development of plants and animals. Moreover, it is also commonly used in production of mineral fertilizers and feed. Although it is an element essential for the life and proper functioning of organisms, in excess it can disadvantageously contribute to the significant deterioration of the natural environment. The discharge of industrial waste and wastewater, as well as eluates from agricultural lands to surface and ground water causes many environmental problems responsible for local excess of nitrogen and phosphorus in biosphere. The characteristic and the most visible consequence of excessive local nutrients concentration in surface water is eutrophication phenomenon, i.e., the process of excessive and too rapid development of aquatic plants disrupting the natural bio-balance in the aquatic, leading thus to its systematic deterioration. Due to the relatively low solubility of naturally occurring solid phosphorus compounds, its biogeochemical cycle differs from the typical cycles of other elements. Phosphorus circulates in the natural environment mainly between the hydrosphere and geosphere, where it participates in a series of chemical and phase transformations related to dissolution and precipitation^1,2^.

Water where phosphorus concentration is higher than 0.1 mg/dm^3^ is classified as sensitive to eutrophication^3^. However, the concentration of phosphorus in water reservoirs located in urbanized areas significantly exceeds 0.2 mg/dm^3^^4^. This is closely related to the average concentration of total phosphorus in municipal sewage—6.0 mg/dm^3^ ^5^. Phosphorus in dissolved forms, as suspended solids and as colloids, occurs in aqueous systems in the form of orthophosphates, pyrophosphates and organically bound phosphorus, thus total phosphorus concentration is represented by the sum of these three phosphorus forms contents. The largest group is represented by phosphates (orthophosphates). These can be identified mainly as different ionic forms, depending on environment pH. For pH below 6, H_2_PO_4_^-^ ions predominate, at pH above 6 the HPO_4_^2-^ ions are mainly present, while for pH higher than 9, PO_4_^3-^ ions dominate. The most common polyphosphates in water and wastewater are pyrophosphates, tripolyphosphates and metaphosphates, whereas the most common organic phosphates are phospholipids, phosphoramides, nucleotides and sugar phosphates^6^.

Precipitation methods are commonly used in water and wastewater treatment technologies to remove various pollutants as sparingly soluble solids. When crystalline phase is demanded, of required size distribution, shape, etc., more advanced technical methods should be applied like reaction crystallization processes^7–10^. In the coagulation process, after adding coagulants (aluminium or iron compounds) to wastewater, reaction with phosphates(V) dissolved in water occurs forming insoluble, sedimenting compounds, thus approximately 60% of phosphorus compounds can be removed^6^. Addition of lime or magnesium chloride with sodium hydroxide results in the precipitation of calcium or magnesium phosphate(V) salts. For example, the three-stage struvite precipitation in a fluidized bed provides 94% efficiency in reducing phosphorus concentration in purified aqueous system^11^. Other technologically efficient techniques used to separate phosphorus from aqueous systems include ion exchange (REM-NUT process) – with efficiency of 93% or adsorption in column beds, like on ceramic granulate of LECA type expanded clay – with P separation efficiency of 88%^12^. Other adsorbents which may be applied in phosphorus removal, include active carbons^13^, metal oxides^14,15^ and aluminosilicates, e.g. bentonite modified with lanthanum(III) (commercially named *Phoslock*) or La-modified ZSM-5 zeolite^16,17^. A new solution to improving the quality of natural waters is the usage of commonly available and cheap biopolymers, for example, cellulose, pectin, alginate or chitosan^18,19^. Such an example of an adsorbent are composites consisting of lanthanum compounds, bentonite and chitosan, which possess very good parameters of the adsorption^20^.

Chitosan is the most important chemical derivative of chitin, the second polysaccharide most commonly occurring naturally in biosphere—after cellulose. Chitosan is characterized by good moisture adsorption characteristics and slow release of adsorbate. It is a widely available and inexpensive biopolymer used in wastewater treatment and in agriculture, mostly as a component of membrane materials^21–23^. Chitosan is also used in removal of metal ions from their aqueous solutions owing to the presence of surface amine and hydroxyl groups acting as chelation sites. Furthermore, chitosan is characterized by its ease to establish various morphological structures, such as films, nanofibers, hydrogels, nanoparticles, and microspheres^24–28^. A particularly interesting chitosan property is the ability to be modified physically or chemically. As a result of the modification, it is characterized by higher mechanical strength and better resistance to changeable chemical conditions^29^.

As early as in 1998, chitosan resins were started to be studied as chemically modified matrices^30–33^. Currently, modification of the chitosan skeleton is done to produce adsorbent with controllable surface morphology. Modifications with carbon materials, e.g., activated carbons, biochars, graphene and nanotubes are already described in the literature^24,34^. Modifications with zeolites and aluminosilicates mentioned above are also known^20,35–37^. In recent years, there have been many papers describing research on the recovery of phosphates(V) on sorbents with particles of magnetic oxides, mainly employing Fe_3_O_4_ nanoparticles^38,39^.

One convenient form of chitosan for the adsorption process is a hydrogel. The application of chitosan in the hydrogel form makes more convenient separation of the used adsorbent from the solution possible, as well as it contributes to the improvement of the ion’s penetration into the inner adsorbent structure, where the adsorption centres are mainly located^34,40^. The use of cross-linked hydrogel provides its stability at pH 3, recommended for the adsorption of orthophosphates from aqueous solutions^41^. A significant advantage of using chitosan hydrogels as adsorbents in the natural environment problems is their biocompatibility and biodegradability, as well as the ability of the chitosan matrix to be modified with metal compounds^42,43^.

In recent years, the use of rare earth elements in wastewater treatment becomes more common^14,15^. The Ce(IV) gains particular attention due to its strong oxidation capacity under acidic conditions^44,45^. Cerium(IV) compounds proved to be effective in oxidizing various organic substances^46,47^, contributing to decolorization and stripping odours from wastewaters, reduction of water turbidity, and even lowering the concentration of arsenic, fluorine and cadmium species in aqueous systems^48^. Currently, cerium and other rare earth metals compounds are used to modify biopolymers towards desired properties and to increase their efficiency in removing various contaminants^49–51^.

The work presents an innovative technological method of phosphorus compounds removal from aqueous systems based on its adsorption on chitosan-based hydrogel with a dispersed cerium(IV) oxide, which demonstrates relatively high specific surface area and improved phosphates(V) adsorption ability.

From the scientific point of view, it is a completely new physicochemical system, thus determining the possibility of obtaining a number of new physicochemical data regarding the behaviour of the system defined in this way in various process (application) environments.

The query of the available scientific and technical literature shows that the presented chitosan-cerium(IV) complex adsorbent system is presented for the first time in the literature, thus providing completely new and original measurement data.

The presented preliminary measurement data related to the application of this new system—as an innovative adsorbent for phosphate(V) ions from aqueous solutions, not yet described in the literature—clearly suggest that this system allows for several clear technological benefits related to increased separation capabilities of phosphate(V) ions in relation to the data presented in the literature.

## Results and discussion

### Ce-CTS adsorbent characteristics

#### FTIR

**Figure 1:**
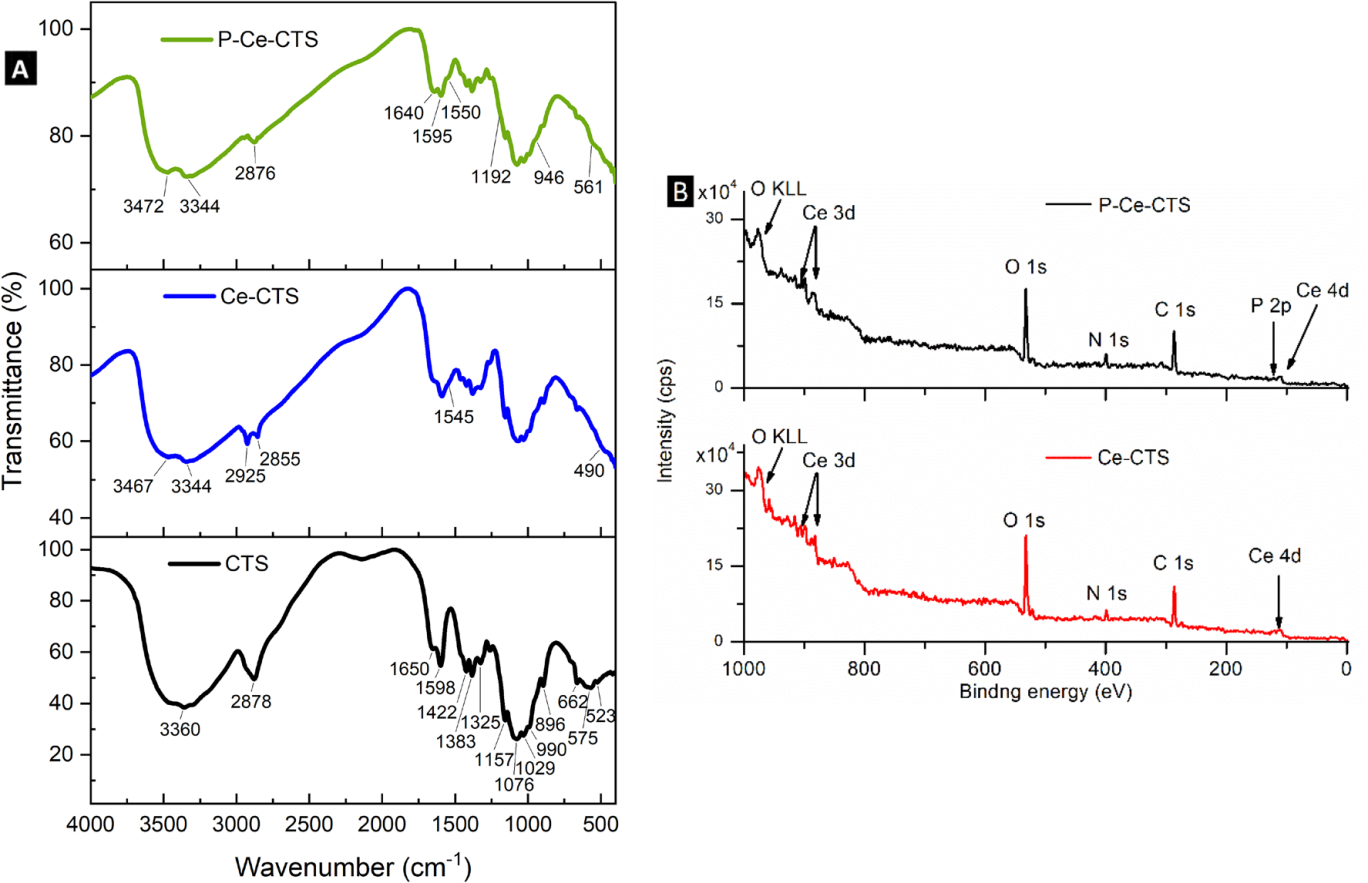
FTIR spectra of the chitosan-based adsorbent (CTS), modified with cerium (Ce-CTS), modified with cerium after phosphates(V) adsorption (P-Ce-CTS) (**A**) and XPS spectra of the chitosan-based adsorbent modified with cerium (Ce-CTS) before (**B**-bottom panel) and after phosphates(V) adsorption (**B**-top panel).

The spectrum of the non-cross-linked chitosan particles – Fig. 1A (left panel, CTS curve) showed characteristic peaks of the amide I band from non-deacetylated amine groups (C=O stretching) at 1650 cm^-1^, amide II (N–H bending overlapped by the amide I band) at 1598 cm^-1^, a complex C-H skeletal deformation –CH_2_ wagging coupled with -OH in plane deformation at 1422, 1383, 1325 and 1261 cm^-1^, ether group (C–O–C stretching) at 1157 cm^-1^, complex overlapped signal between 1100 and 900 cm^-1^ with four maxima at: 1076, 1029, 990 and 896 cm^-1^ from secondary and primary hydroxyl groups (C–OH stretching) and primary amine groups (C–NH_2_ stretching), carbon ring breathing mode and the -CH bending out of the plane of chitosan saccharide ring. The peaks at: 662, 595, 575, 566 and 523 cm^-1^ represent the several out-of-plane bending modes of –NH and C–OH groups. At 2878 cm^-1^ the visibly broad peak is observed of C–H stretching from different –CH_2_ and –CH fragments present in the chitosan structure. The combined broad signal with a maximum at 3360 cm^-1^ presents the stretching vibration of hydrogen connected with heteroatoms, i.e., from several –O–H groups of chitosan, deacylated free –N–H_2_ group and –N–H from acetylated units^52,53^.

In the case of chitosan-based adsorbent loaded with in situ prepared cerium dioxide the typical signal of the chitosan matrix is observed—Fig. 1A (left panel, Ce-CTS curve). The presence of ceria due to the interaction with –OH and –NH_2_ groups, leads to the clearly distinguishable two broad signals from hydrogen-heteroatom stretching with a maximum located at 3467 and 3344 cm^-1^, correspondingly. The CeO_2_ structure is not fully crystalline and probably not fully dehydrated. Thus, it may contain a partially amorphous phase that is connected with the synthesis route. The cerium salt hydrolysis and ceria precursor formation were done in situ in chitosan hydrogel beads and neither further annealing and/or calcination were performed. In such cases, the presence of surface –OH and –OOH groups are expected and the peaks of -OH and -NH_2_ stretching vibrations are even broader than in the spectrum of non-cross-linked chitosan, as well as adsorbed water molecules are visible in Ce-CTS spectrum^54^. The evidence of the presence of different forms of ceria in Ce-CTS is the change of the FTIR spectrum in the range of 400–750 cm^-1^. In this region, several asymmetric and symmetric Ce–O stretching of ceria and hydrated ceria appear, and only one signal of chitosan at 663 cm^-1^ is still noticeably in the declining slope^55–57^. The broadening and change in intensities of amide I, amide II and -OH functional groups in plane deformation signals imply a direct coupling (coordination) of ceria with chitosan’s amine and hydroxide groups. Besides the broadening, also a new peak at 1545 cm^-1^ appears in the amide band region.

After phosphate(V) anions adsorption (P-Ce-CTS curve in Fig. 1A–left panel) the hydrogen-heteroatom stretching vibrations beyond 3000 cm^-1^ were further separated into two peaks with a slightly shifted maxima at 3472 and 3341 cm^-1^, respectively. The signals of tetrahedral phosphate(V) triple degenerated, stretching and wagging in the shape of the slope are noticeable at 1192 and 561 cm^-1^, appropriately. The weaker non-degenerated stretching around 950 cm^-1^ and P=O asymmetric stretching around 1370 cm^-1^ are not visible^58,59^. The more pronounced are the changes in relative intensity of amide bands at 1640, 1595 and 1550 cm^-1^. Their broadening means that these parts of chitosan are involved in the adsorption of phosphates(V) through the interaction between nitrogen-phosphorus or nitrogen-cerium-phosphorus.

The assignment of the most important vibration modes appearing in plain CTS beads, ceria loaded, and after phosphate(V) sorption are listed in Table 1.

**Table 1:** Vibration modes of plain CTS, Ce-CTS and P-Ce-CTS adsorbents.

3360 cm^-1^	–O–H, –N–H combined stretching
2878 cm^-1^ broad	C–H stretching of –CH_2_ and –CH
1650 cm^-1^	C=O stretching, amide I band
1598 cm^-1^	N–H bending, amide II (overlapping with amide I)
1422 cm^-1^	C–H skeletal deformation
1383 cm^-1^	–CH_2_ wagging
1370 cm^-1^ weak	P = O asymmetric stretching
1325 cm^-1^and 1261 cm^-1^	–OH in plane deformation
1192 cm^-1^	tetrahedral phosphate(V) triple degenerated stretching
1157 cm^-1^	C–O–C stretching in ether group
1076 cm^-1^, 1029 cm^-1^, 990 cm^-1^ and 896 cm^-1^	C–OH and C–NH_2_ stretching of primary amine groups and primary& secondary hydroxyl groups C–C ring breathing mode –C–H bending out of the plane of saccharide ring
946 cm^-1^ weak	tetrahedral phosphate(V) non-degenerated stretching
750–400 cm^-1^	asymmetric and symmetric Ce–O stretching
662 cm^-1^, 595 cm^-1^, 575 cm^-1^, 566 cm^-1^ and 523 cm^-1^	–N–H and –O–H out-of-plane bending modes
561 cm^-1^	tetrahedral phosphate(V) triple degenerated wagging

#### XPS

Figure 1B (right panels) presents survey of XPS spectra recorded for both examined samples, chitosan-based hydrogels modified with cerium (Ce-CTS) before and after phosphates(V) adsorption (P-Ce-CTS). The results clearly demonstrate the existence of expected main energy regions, i.e., C 1 s, N 1 s and Ce 3d (with slight indication of secondary Ce 4d region). Next, the O 1 s energy region is visible (with accompanying parasitic Auger line O KLL) and very slight contribution of P 2p energy region in the vicinity of Ce 4d is observed.

**Figure 2:**
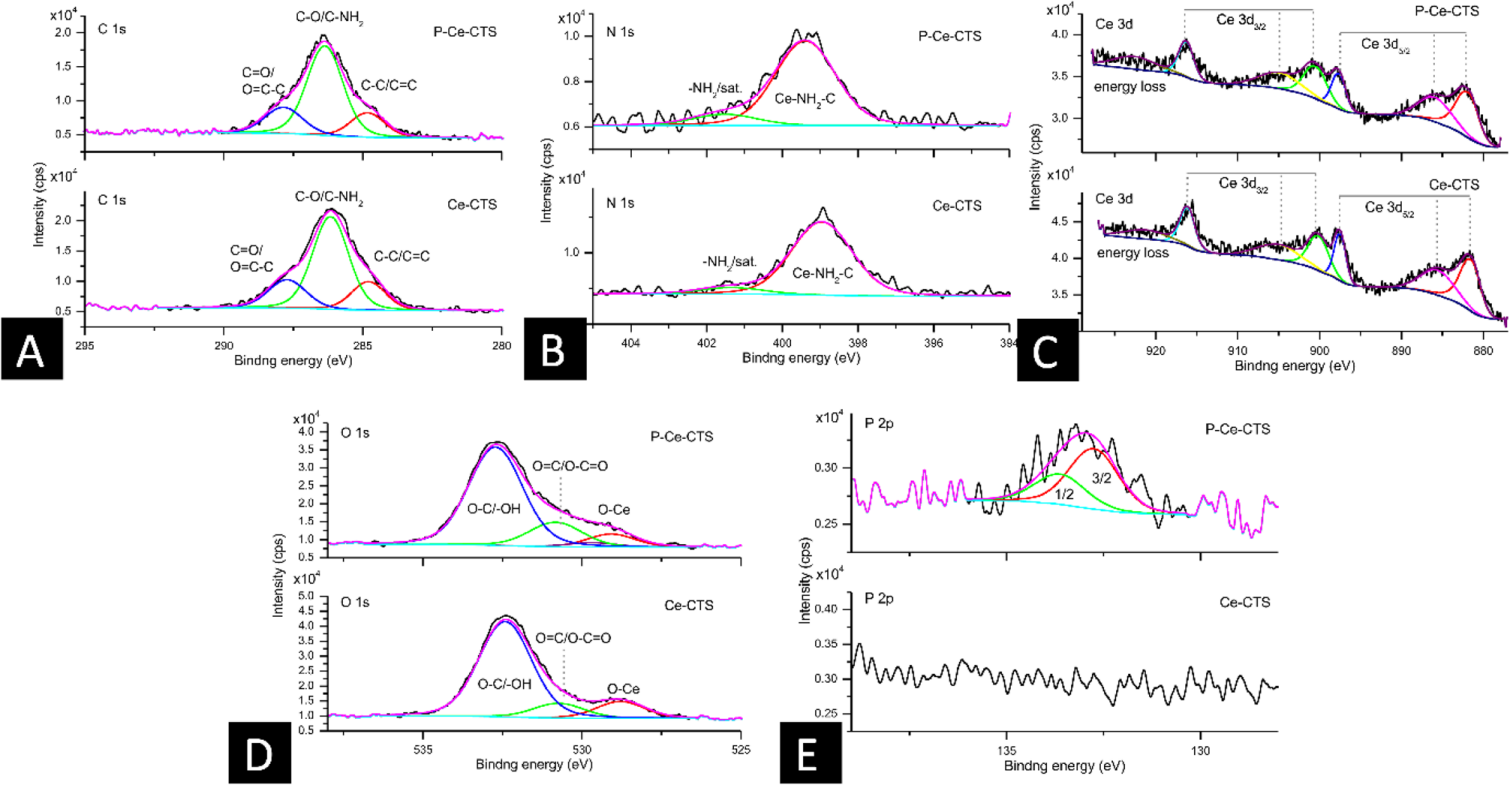
The results of high-resolution XPS scans for Ce-CTS (bottom panels) and P-Ce-CTS (top panels) for C 1 s energy region (**A**), N 1 s energy region (**B**), Ce 3d energy region (**C**), O 1 s energy region (**D**) and P 2p energy region (**E**).

The significant oxygen presence suggests that, besides chitosan-related contribution, either cerium is oxidized or there is identified an extensive oxygen—related species adsorption at the composite surface. For detailed analysis what is the most probable cerium bonding mechanism, the detailed high-resolution spectra were recorded, presented in Fig. 2.

Figure 2A shows the C 1 s energy region for Ce-CTS (bottom panel) and chitosan-based adsorbent modified with cerium after phosphates(V) adsorption – P-Ce-CTS (top panel). The majority of the signal seems to be unaltered after modification. The signal decomposition reveals three distinct components which can be ascribed to C–C/C=C configuration (at 284.8 eV); to C–O/C–NH_2_/C–O–C configuration (the most intense peak, at ~ 285.2 eV) and C=O/O=C–C configuration (at ~ 287.8 eV)^60^.

Further, N 1 s energy region was recorded (Fig. 2B) for Ce-CTS (bottom panel) and P-Ce-CTS (top panel), respectively. In both cases two components can be distinguished out of which first one (at ~ 399 eV) shall be ascribed to Ce–NH_2_–C configuration^49^ while the second is most likely NH_2_ component satellite feature. The position of the first component is, however, relatively high in BE scale taking into advance the electronegativity of Ce (1.12 vs 3.04 of N). This fact could indicate, that the Ce-NH_2_ is not a strong chemical bonding but a kind of coordination or physical bonding only.

Also the Ce 3d region was analysed, as shown in Fig. 2C for Ce-CTS (bottom panel) and P-Ce-CTS (top panel). The both spectra show classical for Ce(IV) formation with respective Ce 3d_5/2_ and Ce 3d5_3/2_ spin–orbit splitting of 18.6 eV^61^. As a result one can observe six distinct components representing one chemical state as a consequence that each spin–orbit component is further split by respective multiplet^62^. The lack of any additional component existence supports the statement revealed from N 1 s analysis that there is no strong chemical bonding between Ce and N. The second consequence would be that CeO_2_ is the dominating form of Ce(IV). In case of P-Ce-CTS sample, there is however, a slight divergence; the main component (at ~ 882 eV) is slightly broadened and shifted with ca. 0.4 eV towards higher binding energy range. One can interpret this as a consequence of phosphorus addition to the sample interaction. However, since the decomposition of Ce(IV) oxide is unlikely (the classical oxide multiplet formation is conserved and there is no trace of additional chemical-bonding originating component in the spectrum) again the coordination–like interaction should be considered. Additional insight may be given by O 1 s analysis for Ce-CTS (Fig. 2D–bottom panel) and P-Ce-CTS (Fig. 2D–top panel). In both cases one can distinguish three main components that can be ascribed to O-Ce configuration (at ~ 529 eV), to O=C/O–C=O at ~ 531 eV and to O–C/–OH at ~ 532.5 eV. While the first one confirms the existence of cerium oxide, the latter two are expected due to chitosan composition. In the case of P-Ce-CTS sample there is, however, the divergence. At ~ 529.5 eV additional component can be detected which is most likely the phosphorus–based O-P configuration^60^.

To check the latter, the P 2p energy region was investigated. The results, shown in Fig. 2E for Ce-CTS (bottom panel) and P-Ce-CST (top panel) show the lack of phosphorus signal in case of Ce-CTS and one configuration with 0.87 eV spin–orbit splitting in case of P-Ce-CTS sample^63^. One can expect the classical PO_4_ (expected at 134 eV signal) but what one gets is nearly 133 eV which is close to some metal phosphate(V) form, e.g., cerium phosphate(V)^60^. This indicates clearly the Ce impact on the P, as well as this can be interpreted as an explanation that this P–related component in O 1 s is at lower binding energy position compared to classical PO_4_^3-^ groups.

## SEM–EDS

**Figure 3:**
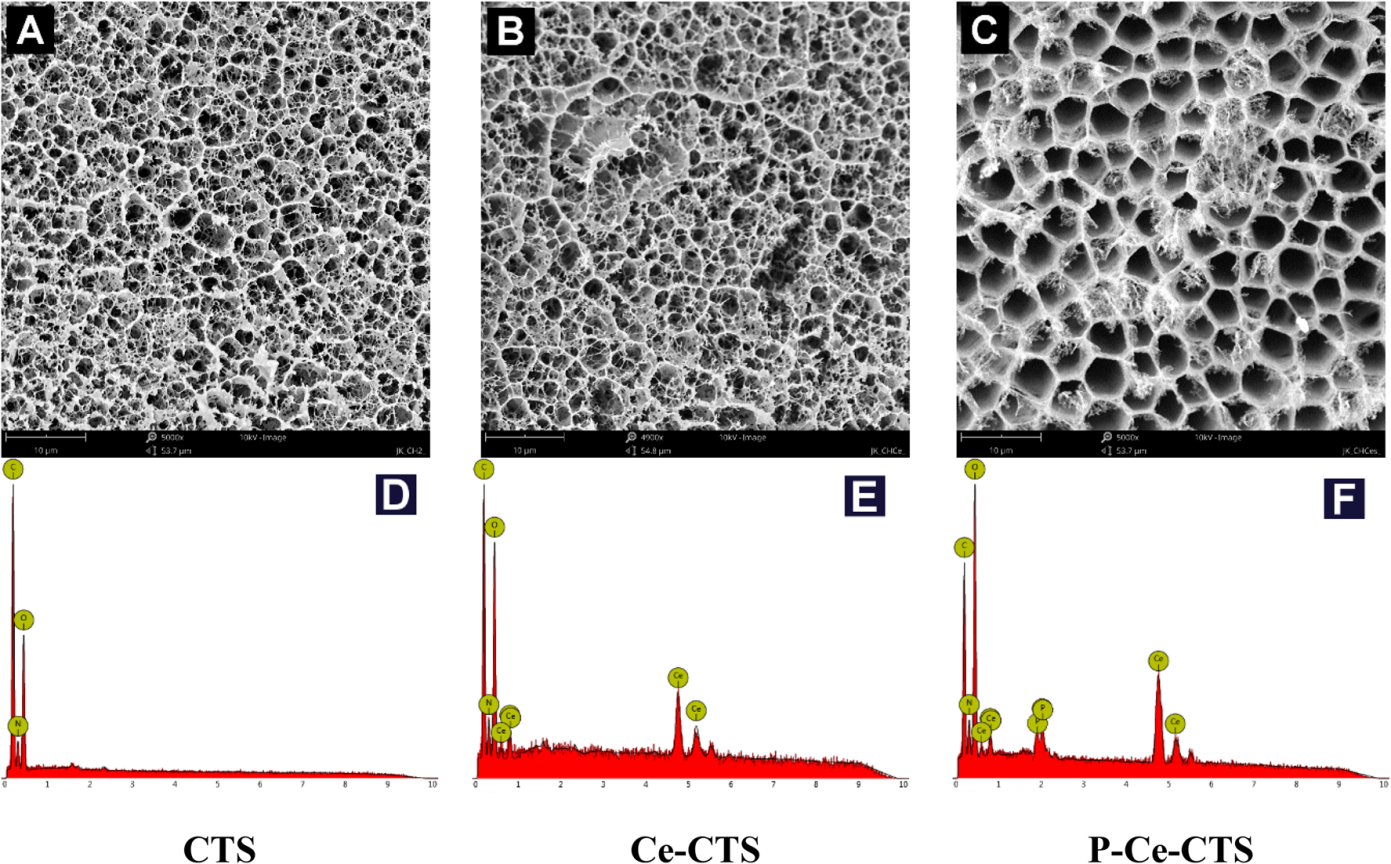
SEM images of chitosan-based hydrogel (**A**), hydrogel modified with cerium(IV) (**B**), hydrogel modified with cerium(IV) after adsorption of phosphates(V) (magnification 5000×) (**C**) and the exemplary corresponding EDS spectra (**D**–**F**).

Analysis of the morphology of freeze-dried chitosan-based hydrogel (CTS) and cerium-modified chitosan-based hydrogels (as composite adsorbents) before (Ce-CTS) and after phosphate(V) ions adsorption (P-Ce-CTS) performed by scanning electron microscopy (SEM) are shown in Fig. 3A–C. From the figure it can be seen that the prepared chitosan-based hydrogels form a quite-well defined and highly porous 3D network, with a characteristic interconnections between the pores. The pore sizes range from micrometer up to dozen micrometers. It could be noticed that the presence of cerium and the phosphate(V) ions adsorption process have an insignificant influence on the developed polymer network structure, its morphology and internal porosity. Both non-cross-linked and cerium-modified hydrogels demonstrate even similar pore system morphology. The overall pore size distribution of cerium-modified chitosan-based hydrogels before and after phosphate(V) ions adsorption actually does not change and still demonstrates the similar three-dimensional network structure. The population of smaller and larger pores is not numerous, so the average diameters of the pores are between 6 and 7 µm and are comparable considering that the differences are within the estimation error limits (Table S1). Generally, no clusters and agglomerates of cerium or phosphates(V) species are visible, so these are homogeneously distributed within the hydrogel bulk volume. The pores are not, however, present on the bead surface. The surface layer forms a wrinkled maze-like structure that may result from the pressure change due to the outflow of water vapor during a freeze-drying process (Fig. S1).

In addition to imaging the hydrogel structure, EDS analysis was also carried out, which confirmed the incorporation of cerium into hydrogel network (Fig. 3E) and adsorption of phosphates(V) (Fig. 3F). The appearance of new bands at: 0.68, 0.88, 0.90, 4.83, 4.84, 5.26 and 5.61 eV corresponding to cerium Mz, Ma, Mb, La2, La1, Lb1 and Lb2 lines, respectively, is noticed in Fig. 3E comparing to Fig. 3D. In case of phosphorus the appearance of band at 2.01 eV (Ka1&Ka2) and 2.13 eV (Kb1&Kb3), as well as the increase in the intensity of the oxygen Ka1&Ka2 lines at 0.52 eV are observed. The average cerium and phosphorus content estimated from the EDS analysis in over a dozen individual sampling points are equal to 48.26 ± 3.26 wt% and 3.48 ± 0.19 wt%, appropriately. The slight increase in the percentage of nitrogen and oxygen in the hydrogel was also observed due to the use of cerium(IV) nitrate(V) as a ceria precursor.

### XRD

Figure S2 (in Supplementary Information) presents the results of the XRD analysis of the non-cross-linked CTS and cerium-modified chitosan-based adsorbents before and after phosphate(V) ions adsorption. Chitosan (A curve in Fig. S2) presents an amorphous phase. A characteristic bump between 10° and 20° of 2 Theta is visible in the two samples with chitosan-based composition^64^. XRD analysis showed that chitosan-based adsorbents after modification (B and C XRD patterns) consist of cerium(IV) oxide (CeO_2_). The registered peak positions at 28.5°, 33.0°, 45.7° and 55.5° of 2 Theta correspond to CeO_2_, mineral cerianite (PDF card no: 04-011-8994). Similar results were presented in other work^65^, where chitosan was modified by cerium compounds to obtain a bio-nanomaterial. No significant differences in the XRD patterns were observed between the chitosan-based adsorbents—before and after phosphate(V) ions adsorption due to formation of the amorphous phase.

## Phosphate(V) ions adsorption

### Effect of solution’s pH

The speciation of phosphates(V), as mentioned earlier, depends on the solution pH. The pH parameter, in addition to influencing speciation, affects also the mechanism and effectiveness of the adsorption process itself^66^.

**Figure 4:**
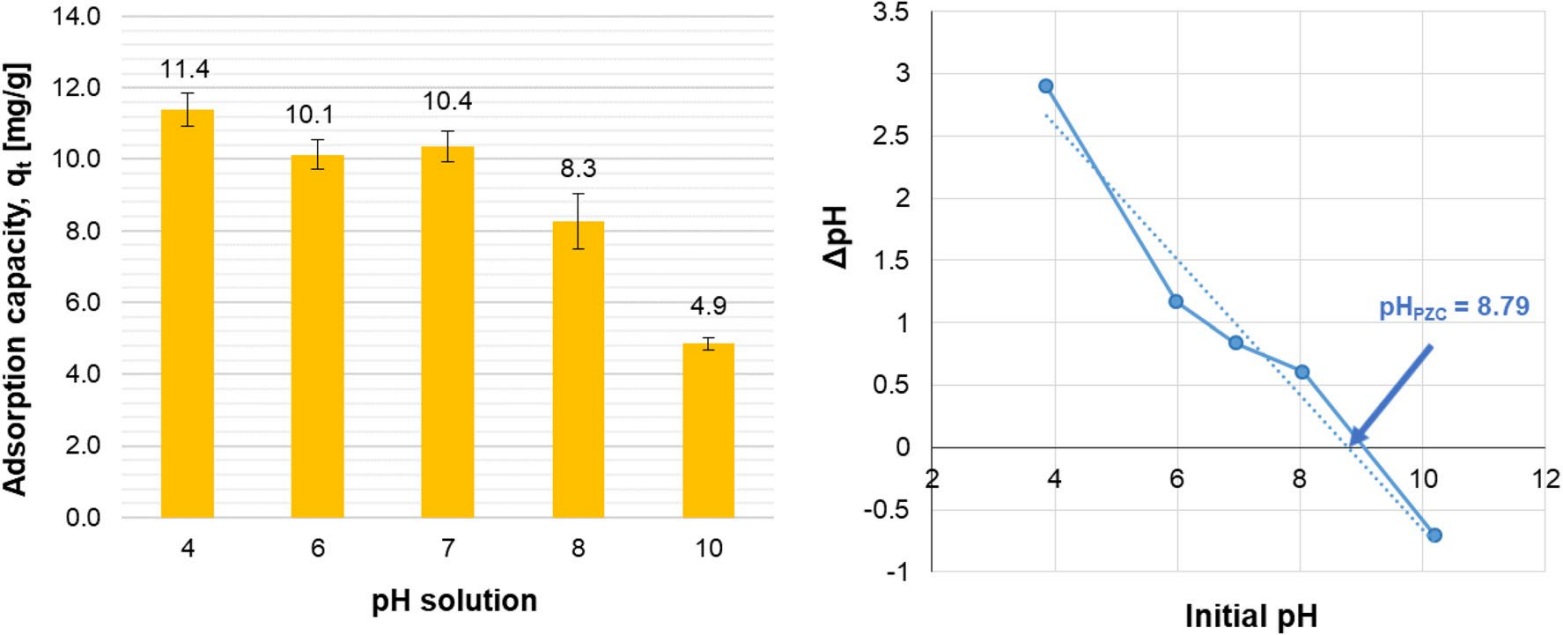
Effect of pH solution (left panel) and pH_PZC_ diagram (right panel) for the cerium-modified chitosan-based adsorbent – experimental data. Ce-CTS (20% wt. of cerium) hydrogel dose: 20 g/dm^3^ (0.8 g/dm^3^ for dry mass); initial concentration of P-PO_4_: 9.3 ± 0.1 mg/dm^3^; contact time: 48 h; temperature: 20 ± 1 °C, tested pH range: 4–10. The bars represent the RSD for two repeats.

In the presented experiments, the phosphates(V) removal from aqueous solutions with the use of the cerium-modified chitosan-based hydrogel was tested for different pH values. From the data obtained (Fig. 4–left panel) it results, that a slight decrease in the adsorption capacity (Eq. (S1)) in Supplementary Information) is observed for pH between 4 and 7. This may be interpreted as a partial change of the phosphorus speciation form—from H_2_PO_4_^-^ to HPO_4_^2-^ corresponding to its lower adsorbability on the tested hydrogel structure. Despite change in pH, the effectiveness of phosphorus compounds removal in such conditions was still high, around 90%. The phosphates(V) adsorption capacity decreased, however, with further increase in pH, and at pH 10 it is almost 40% of the effect corresponding to pH 4. Similar observations were also reported by other researchers. The non-crosslinked chitosan hydrogel showed the highest adsorption efficiency at pH 4 while the chitosan hydrogel crosslinked with epichlorohydrin or polyethylene glycol/chitosan or polyvinyl alcohol/chitosan were the most effective in binding phosphates(V) at pH 3^30,41^. In turn, removal of phosphate(V) ions from aqueous systems using the chitosan-based hydrogel modified with metal ions, like e.g. zirconium(IV), zinc(II), lanthanium(III) ions, demonstrated optimum performance at pH around 4—similar to research effects corresponding to Ce-CTS hydrogel^50,67,68^. Furthermore, at lower pH values (ca. pH < 3), the hydrogel tested swelled and partly dissolved, losing thus its ability to bind phosphates(V). It can be clearly seen that the chitosan-based hydrogel shows high phosphorus (as phosphates(V)) removal rate regardless of pH 4. However, the phosphates(V) removal effect is only slightly lower at neutral pH (ca. 7). This provides more practical possibilities, for example, in the treatment of natural waters, usually of pH ca. 7.

Point of zero charge on a surface (pH_pzc_) of Ce-CTS hydrogel was 8.76 (Fig. 4–right panel). This pH_pzc_ physicochemical parameter value is interpreted such that the surface of the adsorbent had a positive charge below pH 8.76 and a negative charge above this value. The positively charged hydrogel surface promotes the process of phosphates(V) adsorption, which at pH 4–7 occurs in the form of one and two negative anions and could be electrostatically attracted to the hydrogel surface.

### Adsorbent composition

**Figure 5:**
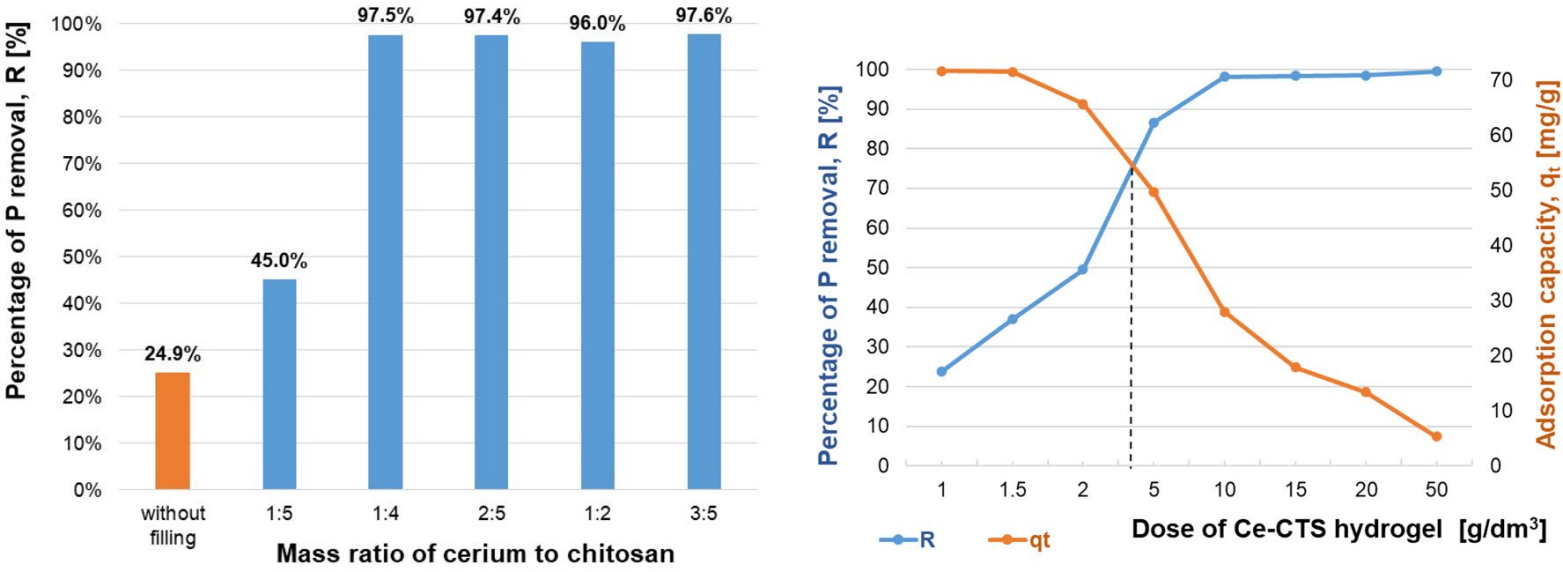
Dependence of the efficiency of phosphates(V) removal from their aqueous solutions on the mass of cerium(IV) dispersed phase used in the chitosan-based composite hydrogel of 20 g/dm^3^ dose (left panel) and on the dose of chitosan-based composite hydrogel modified with cerium(IV) ions in the ratio of 1:4 (right panel). Initial concentration of P-PO_4_: 9.3 ± 0.1 mg/dm^3^; contact time: 48 h; temperature: 20 ± 1 °C.

Due to the unit cost of the dispersed phase introduced into the chitosan-based hydrogel (as cerium salt) and its low mineral resources in the geological environment^69^, it becomes important to determine its optimum dose for the phosphates(V) adsorption process. The present study included six types of such adsorbents: hydrogel without dispersed phase introduced (non-cross-linked CTS) and composite hydrogels with dispersed phase, where the mass ratio of cerium to chitosan was: 1:5, 1:4, 2:5, 2:4 and 3:5, respectively. From Fig. 5 (left panel) it can be seen that the adsorbent CTS demonstrated the lowest efficiency of phosphates(V) removal (Eq. (S2) in Supplementary Information). Considering that the similar results corresponded to all composite adsorbents (with dispersed cerium(IV)), it was concluded, that the composite adsorbent with a 1:4 ratio (20 wt. % of cerium salt phase) was optimal (in experimental conditions: 20 g of hydrogel per 1 dm^3^ of solution with a concentration 9.3 ± 0.1 mg/dm^3^ P-PO_4_ and pH = 7). In practice, it means the increasing the percentage of phosphorus removal and adsorption capacity, from 24.9% and 2.1 mg/g up to 97.5% and 11.0 mg/g, respectively. As the optimisation criteria the significant removal effect of phosphates(V) and the lowest material consumption were assumed. Therefore, composite chitosan-based hydrogel containing dispersed cerium(IV) in the ratio of 1:4 to chitosan was used as the further tested samples. Furthermore, no leaching of the cerium ions from the composite into the solutions after adsorption was identified analytically. It means, that the proposed adsorbent was stable under the studied conditions of the phosphate(V) removal process. Due to the lack of leaching of the ceria filling, its potential for use in water purification is becoming more valuable. The use of a relatively expensive element such as one of the lanthanides is compensated for by its lack of loss after adsorption, allowing it to be recycled. The filling is permanently bonded to the chitosan structure, and the introduced modification, in addition to stability, shows increased mechanical durability (the adsorbent does not fall apart and is convenient for manual operation).

### Adsorbent dose

For the tested chitosan-based composite hydrogel modified with cerium(IV) salt, the optimal dose of adsorbent in the suspension was the next process parameter under study. Increasing the adsorbent dosage, one can increase the phosphates(V) removal efficiency since the number of available active adsorption sites rises.

Based on the experimentally identified dependence between the efficiency of phosphates(V) removal and the hydrogel dose (Fig. 5–right panel), it can be concluded, however, that this efficiency stabilized when using 10 g of hydrogel per 1 dm^3^ of the solution, with a concentration 9.3 ± 0.1 mg/dm^3^ P-PO_4_ and at pH 7, on the level of 98.2%. Further increasing the hydrogel adsorbent dose increased the adsorption efficiency only slightly. It should be mentioned here, however, that the concentration of phosphates(V) used in the research significantly exceeded the average level identified in the natural water reservoirs (usually below 0.2 mg/dm^3^). This means that for water reservoirs with a lower concentration of phosphates(V), the hydrogel dose can be reduced. On the other hand, some environmental studies^70^ focused, that in some cases water can be highly contaminated with phosphates(V)–even up to the concentration level investigated in this study (approx. 0.3 mM P-PO_4_).

Moreover, the study proved the high potential of the hydrogel surface for phosphates(V) adsorption, as the use of 1 g of hydrogel per 1 dm^3^ of phosphate(V) solution corresponds to its adsorption capacity of 71.7 mg/g (Fig. 5–right panel). Importantly and advantageously, no disruption of the adsorbent structure or no leaching of the filling from the composite into the solution was observed; ICP OES analysis showed no presence of cerium in the solution after adsorption.

As can be seen from Fig. 5 (right panel), the optimal dose of adsorbent, taking into account both the efficiency of phosphates(V) removal and the adsorption capacity of chitosan-based composite adsorbent, was about 4 g of hydrogel per 1 dm^3^ of phosphate(V) ions aqueous solution. This is illustrated in Fig. 5 (right panel), graphically indicated as the intersection of the efficiency of the phosphates(V) removal line and the adsorption capacity line. It can be also seen that the phosphates(V) removal efficiency is 75% at this point corresponding to the adsorption capacity of 55 mg of P-PO_4_/g of dry adsorbent. The use of 4 g of hydrogel per 1 dm^3^ of phosphates(V) in their aqueous solution might seem not economical in industrial practice. However, it should be borne in mind, that the proposed adsorbent is a hydrogel in which the dry adsorption mass is only a 4.2% fraction while the rest is represented by swelling water. To use of such adsorbent in a natural water reservoir, it is necessary to conduct further and broader research focused not only on the optimization of the adsorption process itself, but also on phosphorus compounds desorption—to recover both phosphorus and then adsorbent in an economical, cyclic operation mode, according to Circular Economy standards.

### Kinetic studies of adsorption

**Figure 6:**
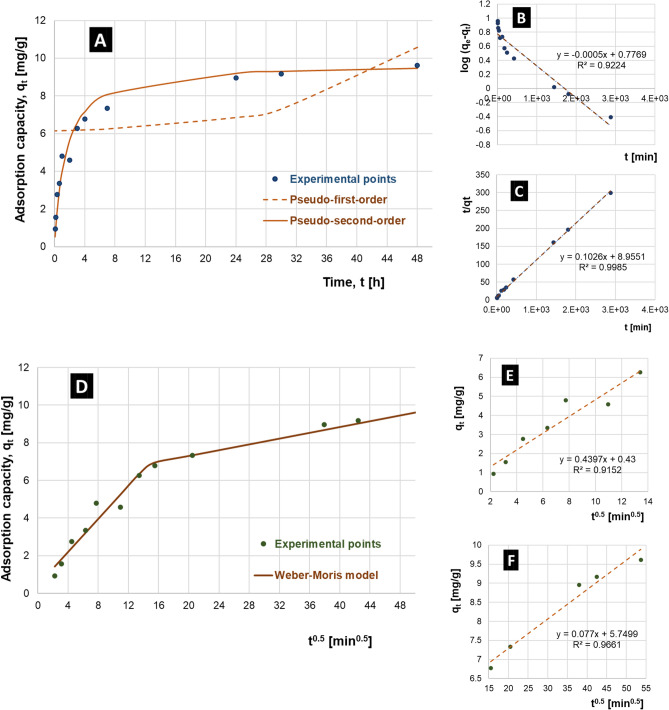
Kinetics of the phosphates(V) adsorption process on the cerium-modified chitosan-based hydrogel—raw experimental data (**A**), the pseudo-first-order model approach (in a linearized form) (**B**), pseudo-second-order model approach (in a linearized form) (**C**), experimental dependence of q_t_ = f(t^0.5^) (**D**), formal Weber-Morris model (in a linearized form) (**E**) and Weber-Morris model in intraparticle diffusion range (in a linearized form) (**F**). Ce-CTS (20% wt. of cerium) hydrogel dose: 20 g/dm^3^ (0.8 g/dm^3^ for dry mass); initial concentration of P-PO_4_: 9.76 ± 0.09 mg/dm^3^; pH: 7; temperature: 20 ± 1 °C, tested contact time: 5–2880 min.

Analysis of the course of the adsorption process in time at pH 7 usually observed in surface waters^71^ and at ambient temperature T = 20 °C, with the initial phosphorus concentration of 9.76 ± 0.09 mg/dm^3^ P-PO_4_ showed (Fig. 6–left panel), that the adsorption capacity of phosphates(V) on the cerium-modified chitosan-based adsorbent (Ce-CTS) increases rapidly during the first several hours of interphase contact, and then the ultimate equilibrium state is slowly approached, reaching it finally—in practice—after ca. 24 h of the process. No practically important changes are observed during the next 24 h of the experiment.

To describe the adsorption process kinetics, two the most often used models with linearized equations were considered^72,73^ a pseudo-first-order kinetic model (Eq. (S3)) and pseudo-second-order kinetic model (Eq. (S4)).

The pseudo-second-order kinetic model (*R*^2^ = 0.9985) shows better agreement with the experimental data than the pseudo-first-order one (*R*^2^ = 0.9224)–see Fig. 6 (right panel). Furthermore, the calculated value of *q*_2_ = 9.75 mg/g agreed well with the experimentally determined adsorption capacity, *q*_t_ = 9.61 ± 0.11 mg/g. The parameter values of both models are presented in Table S2 (see Supplementary Information). Using the calculated kinetic constant *k*_2_ and the equilibrium adsorption capacity *q*_e_, the adsorption rate equation was determined which can be employed in design-oriented calculations concerning the investigated system application in e.g., environmental protection technologies. Furthermore, the results indicated that the chemical reaction may be responsible for controlling the adsorption rate of phosphorus compounds in hydrogel beads as the limiting factor.

In practical application of porous structures of adsorbents, diffusion phenomena may be important from the point of view of mass transport resistances, in particular those related to intraparticle diffusion^74^. In the case of solid–liquid systems, simultaneous occurrence of subsequent diffusion processes is usually observed—external diffusion of the adsorbate in the liquid through the boundary layer, followed by continuation of the diffusion process in the porous structure of the adsorbent (internal diffusion processes) and final surface adsorption phenomenon. Internal diffusion models are represented by the Crank, Weber-Morris and Bangham models^75^. Usually, the Weber-Morris model is used to identify the complex consecutive mechanisms of the process, assuming that intraparticle diffusion is the rate-limiting stage of the entire adsorption process^76^. However, the external diffusion processes are described by the Spahn and Schlunder, as well as Boyd models^75^. The Weber-Morris model is particularly useful for identifying the mechanisms of diffusion processes in relation to the adsorbent-adsorbate system, which cannot be obtained using the pseudo-first order or the pseudo-second order kinetic models alone^77^.

In order to identify the mechanisms of the adsorption process in the investigated system, calculations were carried out using the intraparticle diffusion model by Weber and Morris (Eq. (S5))^78–82^.

The power exponent 0.5 in the formula (S5) results from the theoretical assumptions of the Fick diffusion model and the assumed plate geometry^74^. In the case of the linearity of the q_t_ = *f*(t^0.5^) dependence for all obtained experimental points, intraparticle diffusion processes control the process of adsorbate mass transport, but these are not the only mechanism affecting the observed net course of the process in time^83,84^. Only when the discussed linear q_t_ = *f*(t^0.5^) relationship additionally passes through the origin of the coordinate system (C = 0, which means no or negligibly small diffusion layer thickness in the process system), it can be assumed that the only limitation of the mass exchange rate is intraparticle diffusion (pore diffusion)^78,83^.

The frequently observed successive linear segments of the relationship (Eq. (S5)) for the experimental data correspond to successive stages of mass transfer with gradually decreasing process rates (along with the corresponding kinetic parameters K_i diff_ and C). These can be theoretically interpreted as the processes of external (boundary layer) and internal (diffusion inside a porous particle) mass transport, dependent on the characteristics of the structure (diameter distribution) of macropores, mesopores and micropores, ending with adsorption on the surface of the adsorbent^83^. The values of parameter C in equation (S5) are proportional to the thickness of the boundary layer (and the related diffusion resistance to adsorbate mass transport)^83^.

Based on the value of the rate constant for intraparticle diffusion K_i diff_, the value of D–intraparticle diffusion coefficient–can be determined from the formula (S6). The course of the experimental dependence of q_t_ = f(t^0.5^) is shown in Fig. 6D. In Fig. 6E,F two linear segments can be distinguished (change after 180 min), which proves the applicability of the model(S5). The linear segment not passing through the origin indicates that intraparticle diffusion is one of several mass transport mechanisms in the analysed system. This is not the only mechanism limiting the rate of the adsorption process. Experimental data also clearly indicate a change in the mechanism of the process after 180 min—from diffusion through the film of the surface boundary layer and direct adsorption on the exposed external surface, to the combined mechanism of internal diffusion immediately following this initial stage^75^. Obtained with linear regression methods model parameters are presented in Table S2 (see Supplementary Information)^77,85^.

Considering the significantly higher value of K_i diff (1)_ = 0.43973 mg/(g·min^0.5^) obtained by linear regression (mainly diffusion in the liquid boundary layer and adsorption/surface reaction processes—formal application of model (S5)) compared to the consecutive second linear segment K_i diff (2)_ = 0.07703 mg/(g ∙ min^0.5^) (complex diffusion processes inside the adsorbent particles) it can be assumed, where the intraparticle diffusion mechanism—as a slower process—controls kinetically the overall course of mass transfer in the adsorption process, especially clearly observed after 180 min of the process.

In addition, the inability to identify a larger number of clear linear sub-segments in the intraparticle diffusion range proves that no clear influence of the volumetric pore size distribution on a clear differentiation of the diffusion phenomena rates in this synthetic adsorbent porous structure is observed.

Other available works suggest the interpretation of successive linear segments of the model (S5) as internal diffusion in the adsorbent structure, in macropores, mesopores and micropores, respectively^73^, or as^84^ initial fast surface adsorption taking into account diffusion phenomena in the liquid boundary layer (with possible non-linearity^86^), followed by diffusion in the porous structure of the adsorbent and finally gradual stabilization of the process in the dynamic state of adsorption equilibrium. Two linear segments are usually interpreted as—initially—external diffusion through the liquid boundary layer and adsorption phenomena on the external surface^84,87^, and then as a complex process of internal diffusion in the porous structure of the adsorbent^82,88,89^.

The values of C_1_ and C_2_ indicate a noticeable impact of the diffusion process in the boundary layer—the value of C_2_ = 5.74992 mg/g as significantly higher (over 13-times) than C_1_ = 0.43003 [mg/g] indicates a greater relative influence of the liquid boundary layer for mass transport in the next stage of the process (intraparticle diffusion). A lower C_1_ value suggests relatively easier diffusion in the outer surface layer of the adsorbent.

For the K_i diff (2)_ = 0.07703 mg/(g ∙ min^0.5^) for the second linear segment, corresponding in theoretical interpretation to the phenomena of internal diffusion, the following values of intraparticle diffusion coefficient D were calculated from equation (S6) (considering experimentally identified radius of adsorbent particle grains r = 0.15 cm and experimentally determined adsorption capacity of Ce-CTS adsorbent q_e_ = 9.6 mg/g): D = 1.2635·10^–7^ cm^2^/min]. This is relatively small value of D. For example, the value of intraparticle diffusion coefficient for Ca(II) presented in^83^—for the analysed size of adsorbent particles r = 0.009 cm – is D = 4.60 ∙ 10^–6^ cm^2^/min. Approximately 36-times lower values of the diffusion coefficient obtained in this work can be interpreted in connection with a much (about 17-times) larger radius of the adsorbent particles (r = 0.15 cm), which was responsible for less direct exposure of the more available external surface, and a simultaneous increase in the share of the internal surface of the adsorbent in the mass transfer process, along with the related kinetic limitations resulting from the greater share of internal diffusion transport phenomena effect.

In the work^84^, higher D values were also obtained for the adsorption of malachite green by rattan sawdust—in the 0.44·10^–6^—1.84·10^–6^ cm^2^/min range (r = 0.025–0.050 cm).

### Adsorption equilibrium isotherm studies

**Figure 7:**
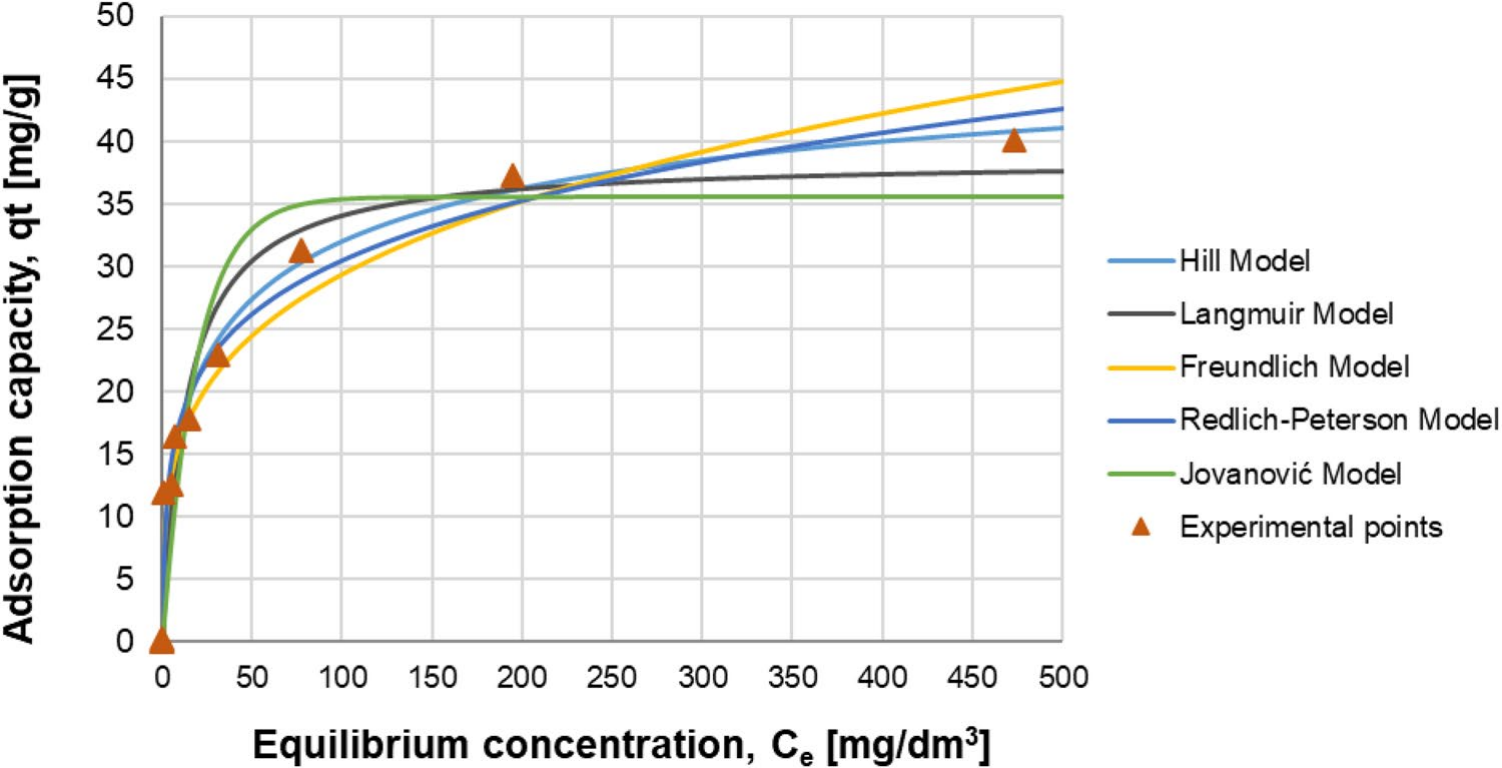
Adsorption isotherm of phosphates(V) on the cerium-modified chitosan-based hydrogel—raw experimental data and five theoretical or empirical adsorption isotherm models. Ce-CTS (20% wt. of cerium) hydrogel dose: 20 g/dm^3^ (0.8 g/dm^3^ for dry mass); pH: 7; contact time: 24 h; temperature: 20 ± 1 °C, tested initial concentration of P-PO_4_: 0.1—500 mg/dm^3^.

Isothermal adsorption equilibrium experiments were studied at a temperature of 20 °C, at pH 7, and in the initial phosphate(V) concentration range of 0.1–500 mg/dm^3^ with the constant dose of chitosan-based adsorbent with 20% wt. cerium(IV) dispersed (1 g per 0.05 dm^3^ of solution) for a contact time of 24 h. The adsorption isotherm shown in Fig. 7 is regular, positive and convex to the concentration axis for Ce-CTS with a steep initial slope, indicating the strong affinity of the phosphate(V) anions to chitosan-based adsorbent. The curve shows a saturation plateau at high phosphate(V) concentration and the experimental adsorption capacity of Ce-CTS achieved a maximal value of 40.1 ± 6.4 mg/g. The isotherm of Ce-CTS can be classified as the L-type according to the classification of Giles et al.^90^.

Analysis of the system in respect to adsorption of phosphate(V) ions from aqueous solutions was carried out using five selected theoretical or empirical models of adsorption isotherms^91–94^ and based on own measurement data.

The Langmuir isotherm model (Eq. (S7) in Supplementary Information) is based on the assumption of dynamic equilibrium, which determines the same rate of adsorption and desorption processes under given conditions. Assuming that adsorption is limited to one surface layer of adsorbate, as well as a constant value of adsorption energy, a defined and evenly distributed number of available energetically identical active sites for adsorption (only one molecule per one site), and the exclusion of potential interactions between already adsorbed molecules—the isotherm Eq. (S7) is obtained. The theoretical assumptions of the model exclude the presence of more molecules in one active site, thus eliminating the potential possibility of many adsorption layers on the available surface of the adsorbent.

The non-linear regression analysis of the experimental data using the Langmuir adsorption isotherm model (Eq. (S7)) provided the following values of the model parameters: *K*_L_ = 0.075 dm^3^/mg, the maximum surface concentration of the adsorbate *q*_m_ = 38.553 mg/g (R^2^ = 0.943).

In the adsorption equilibrium analysis using the Langmuir isotherm model, the *R*_L_ parameter defined by Eq. (S8) is also taken into account. Based on the theoretical predictions, it can be concluded, that for *R*_L_ < 1 there are favourable conditions for the adsorption process, whereas *R*_L_ values close to zero suggest the irreversibility of the process. For *R*_L_ = 1, linearity of the adsorption isotherm is observed, while even higher values of *R*_L_ > 1 indicate unfavourable conditions for the potential adsorption process. In the discussed case, the calculated value of the *R*_L_ parameter = 0.0259 < 1 (for the highest experimentally tested initial concentration of adsorbate in the solution—500 mg/dm^3^), which can be interpreted as theoretically confirmed and observed, practically favourable conditions for adsorption phenomena in the analysed system. It can be assumed, that the presence of cerium dioxide has a certain effect on the formation of the structure of the chitosan network and its intrinsic morphology, influencing the formation of the beneficial structure of the adsorption surface. Neither clusters nor agglomerates of cerium(IV) or phosphate(V) compounds present on the postprocessed adsorbent were observed in the SEM images. However, this indicates that these are uniformly distributed throughout the hydrogel volume, affecting this way the homogeneity of the structure of the adsorption surface (Fig. 3A–C). The *R*_L_ value, which is relatively close to zero, also indicates a process-favourable irreversibility of adsorption phenomena under the analysed process conditions.

Analysis of the process conditions using changes in the value of the *R*_L_ parameter for potentially various values of the highest initial adsorbate concentrations in solution identifies a systematic increase in the value of the *R*_L_ parameter (e.g., for 250 mg/dm^3^ *R*_L_ = 0.0506, 100 mg/dm^3^—0.1176, 15 mg/dm^3^—0.4706, 5 mg/dm^3^—0.7273, 2 mg/dm^3^—0.8696, 0.1 mg/dm^3^—0.9926). Thus, in the entire range of the tested phosphate(V) concentrations, the *R*_L_ values < 1. However, in the range of the smallest solution concentrations, the values of the *R*_L_ parameter–as close to 1–clearly indicate systematically less favourable conditions for adsorption. It should be noted, that for the highest initial concentration values tested, 250–500 mg/dm^3^, the values of the *R*_L_ parameter 0.0506—0.0259 indicate—as close to zero—the technologically advantageous irreversibility of adsorption phenomena. However, this does not exclude the possibility of deliberate desorption of the adsorbate (phosphates(V)) from the adsorbent surface. However, this requires a clear change in the equilibrium conditions in the process, e.g., by appropriate correction of the pH in the desorbing solution (see Fig. 4).

The Freundlich adsorption isotherm model (Eq. (S9)), in contrast to the theory-based Langmuir model (Eq. (S7)), is an empirical equation, particularly useful for describing multilayer adsorption phenomena on heterogeneous adsorbent structures. The model also assumes the possibility of diversifying the surface distribution of latent adsorption heat and the affinity of the adsorbate to this heterogeneous surface.

Analysis of measurement data using non-linear regression methods based on the Freundlich adsorption isotherm model—equation (S9)—allowed to obtain the following model parameters: *K*_F_ = 8.832 (dm^3^/mg)^1/n^, 1/*n* = 0.261 (R^2^ = 0.951). According to the theoretical interpretation, the value 0 < 1/*n* < 1 indicates favourable process conditions, unfavourable for 1/*n* > 1, and the irreversibility of the process in the case of 1/*n* = 1. The obtained value 1/*n* = 0.261 confirms the technologically favourable tested system with regard to the new adsorbent structure and the aqueous solution of phosphates(V). In particular, a more accurate analysis taking directly into account the value of *n* indicates, that for *n* = 2–10 good adsorption conditions are observed, for *n* = 1–2 moderately difficult, while *n* below 1 indicates low adsorption possibilities. For the analysed case the value of *n* = 3.826 confirms good physicochemical conditions in the system for the course of the adsorption process. Favourable physicochemical conditions are especially related to the amorphous structure of the hydrogel mentioned above, confirmed by XRD analysis results (Fig. S2) and the incorporation of the CeO_2_ phase into this amorphous structure, which is characterized by a very well-developed specific surface and active centres focused on anions adsorption, due to positive charge of the surface in the expected pH range. Thus, a technologically convenient composite system was obtained, represented by the support (hydrogel), which enables the beneficial and stable development of the active surface represented by the adsorption-active CeO_2_ crystalline phase, actively counteracting the potential adverse phenomena of its aggregation or agglomeration–which could lead to an unfavourable decrease in the area of interfacial contact with the purified aqueous solution of phosphates(V).

The Redlich-Peterson three-parameter adsorption isotherm model–equation (S10), is an intermediate model showing both the characteristics of the Langmuir Eq. (S7) (when *g* is close to 1) and the Freundlich Eq. (S9) (when *g* is close to 0) (hybrid model). Taking into account the structural mathematical (non-linear regression) flexibility of this adsorption isotherm model, it can be used for the systems where the behaviour suggesting the formation of one adsorbate layer on the surface is not strictly observed. The parameters of the Redlich-Peterson isotherm model obtained using non-linear regression are as follows: *K*_RP_ = 20.957 (dm^3^/mg)(mg/g) = (dm^3^/g), *a*_RP_ = 1.711 (dm^3^/mg)^g^, *g* = 0.799 (R^2^ = 0.981). The value of the *g* parameter is close to 1 (as 0.799), indicates thus the model orientation towards the Langmuir adsorption isotherm model rather, based on the formation of one adsorbate layer.

The Jovanović adsorption isotherm model—equation (S11)—is close to the theoretical assumptions of the Langmuir adsorption isotherm model, taking into account not only mechanical interactions between molecules and the adsorbent surface, but also other mechanical interactions between molecules in the contacting liquid phase (subject to both adsorption and desorption) and already adsorbed molecules. Due to the assumption of the maximum monolayer capacity, the obtained asymptotic values of the maximum adsorbate concentration (monolayer) can be directly compared with the values predicted by the Langmuir isotherm model (Eq. (7)). The following values of model (Eq. (S11)) parameters were obtained by non-linear regression: *q*_J_ = 35.594 mg/g (comparable to the value predicted by the Langmuir model, *q*_m_ = 38.553 mg/g), *K*_J_ = 0.053 dm^3^/mg (R^2^ = 0.901).

The Hill adsorption isotherm model—Eq. (S12)—is helpful in identifying differences related to the mutual interactions of adsorbed molecules. The model is based on the assumption that previously adsorbed molecules may potentially have some influence on other adsorption sites located in the adsorbent structure. In the case of the cooperative process, theoretically the value of the parameter *n*_H_ > 1. The following values of the isotherm model (Eq. (12)) parameters were calculated for the analysed system using non-linear regression: *q*_H_ = 52.666 (mg/g)(dm^3^/mg)^nH^, *n*_H_ = 0.511, *K*_H_ = 6.783 (mg/dm^3^)^nH^ (R^2^ = 0.981). The value of *n*_H_ = 0.511 < 1, corresponding to the analysed equilibrium experimental data, indicates thus that adsorption in the studied case is a non-cooperative process.

The adsorption of phosphate(V) anions on the Ce-CTS adsorbent is the result of not only the electrostatic attraction (physical adsorption) between the positively charged surface of the Ce-CTS hydrogel and the negatively charged forms of phosphate(V) anions, but also stronger interactions, such as chemical bonding of the adsorbate with the adsorbent (chemisorption), which was confirmed by the analysis of the IR and XPS spectra (Figs. 1 and 2). Moreover, no additional interaction was identified between the already adsorbed phosphate(V) complex ions and other adsorption sites on the hydrogel surface, which was independently confirmed by the constant of the Hill adsorption isotherm model (*n*_H_ = 0.511).

The best agreement between the measurement data and the modelling results was obtained for the Hill isotherm model (see Fig. 7 and Table S3), which forecasts the maximum adsorption capacity of ca. 52.7 mg/g on the basis of the available equilibrium data course. The Langmuir and Jovanović isotherm models slightly underestimate the asymptotic value corresponding to the maximum adsorption capacity (*q*_m_ = 38.553 mg/g, *q*_J_ = 35.594 mg/g, correspondingly). In the case of the Freundlich and Redlich-Peterson isotherm models, these saturation asymptotic values would be, in turn, overestimated (however, in the construction of these models there are no numerical parameters corresponding in interpretation to the horizontal asymptotes of the isotherm function).

### Thermodynamic studies

Apart from the purposeful modification of solution’s pH, the temperature change can also affect the phosphates(V) adsorption efficiency on chitosan-based adsorbent investigated^95^. Thermodynamics of phosphate(V) ions adsorption using a cerium-modified chitosan-based hydrogel was investigated in three different temperature values, namely: 298, 308 and 318 K, with an initial concentration of phosphorus 9.3 ± 0.1 mg/dm^3^ P-PO_4_. As shown in Fig. S3 (in Supplementary Information), the adsorption ability of phosphates(V) slightly decreased with the suspension temperature increase. This can be interpreted as a reverse linear dependence between temperature and adsorption capacity and confirms–as expected from theory—that adsorption is an exothermic process for which the temperature increase is unfavourable. To determine the change in adsorption capacity in respect to the temperature change, the basic thermodynamic parameters of this process were determined, i.e. enthalpy, entropy and free Gibbs energy of the adsorption for a chitosan-based hydrogel composite resulting from its modification with Ce(IV) ions (see Eqs. (S13) and (S14) in Supplementary Information)^96^.

In Fig. S3 (right panel) the linear relationship between the natural logarithm of the equilibrium constant *ln* K_c_ and 1/T was presented and evaluated statistically using linear regression method. The high value of the correlation coefficient (R^2^ = 0.9997) confirms the applicability of the model considered to the analysed system. The entropy ΔS^ɵ^ and enthalpy ΔH^ɵ^ values were determined from the regression coefficients (after appropriate theoretical interpretation of the model constants) and are presented in Supplementary Information in Table S4. The negative value of ΔH^ɵ^ = −4.20 kJ/mol indicates, that phosphates(V) adsorption onto hydrogel surface (developed mainly within the internal pores) was exothermal, so the temperature decrease had a positive effect on the adsorption process efficiency and followed the physical adsorption rather than a chemisorption mechanism. The value of ∆H^ɵ^ parameter calculated based on the experimental data was definitely higher than −40 kJ/mol, confirming independently that in this case adsorption was physical in nature, and therefore included weak intermolecular interactions, e.g., of the van der Waals type^97^.

Table S4 shows that the adsorption Gibbs free energy (ΔG^ɵ^) had negative value for all temperatures tested. This indicates a spontaneous process of phosphate(V) ions adsorption on Ce-CTS hydrogel surface. The increase in ΔG^ɵ^ value (closer to 0) with increasing temperature indicated a more intensive adsorption at lower than at higher temperature range. Moreover, the determined Gibbs free energy value determines the nature of the adsorption process under the assumed experimental conditions. The ∆G^ɵ^ value above -20 kJ/mol indicates the electrostatic interaction between the active sites in the chitosan-based hydrogel structure and the phosphate(V) ions, while the value below -20 kJ/mol theoretically suggests the complexation reaction, i.e., charge transfer or its sharing between the adsorbent surface (functional groups) and the adsorbate^98^.

According to Table S4 also the adsorption entropy change demonstrates a negative value (∆S^ɵ^ = -4.83 ·10^–3^ kJ/(mol·K)) in the whole investigated temperature range 298–318 K. Of course, the negative ΔS^ɵ^ is unfavourable for spontaneous adsorption, in opposite to the negative ΔH^ɵ^ and ΔG^ɵ^ values that are favourable for adsorption. Probably, phosphate(V) molecules movable in the solution, were adsorbed in an orderly fashion onto the chitosan composite beads, resulting in entropy decrease. It also means that no significant change occurs in the internal structure of the adsorbent during the adsorption process. Such adsorption phenomena are not favourable at high temperatures. Negative values of *∆S*^ɵ^ and ΔH^ɵ^ also suggest, that the enthalpy effect rather than the entropy change controlled the driving force for the phosphate(V) ions adsorption phenomena^99,100^.

### Comparative studies

A comparison of Ce-CTS adsorbent with the various reported adsorbents are shown in Table 2. A high phosphates(V) adsorption capacity of the studied own adsorbent can be observed compared to other adsorbents described in the literature. In conclusion, the use of the obtained adsorbent for phosphates(V) adsorption, compared to many other adsorbents in the literature, may be suitable for use in, for example, wastewater treatment plants by removing residual phosphorus compounds or for the revitalisation of water bodies.

**Table 2:** Comparison of the phosphates(V) adsorption capacity of various adsorbents (including own results).

Adsorbent type		Maximum adsorption capacity q_m_ [mg/g]	pH/ T [K]	Concentration range [mg/dm^3^]/ adsorbent dose [g/dm^3^]
Chitosan-based adsorbents	Cs-La^50^	107.7	4.0/293	1–100/0.5
	**Ce-CTS (own results)**	72.0*	**7.0/293**	**0.1–500/1**
	Cs-Zr^68^	60.6	4.0/–**	1–40/0.2
	CB-G-Cu^101^	53.6	7.0/295	1–100/1.25
	CHs^102^	39.9	3.0/, –**	10–1000/1
	Cs-Ca^28^	23.7	7.0/298	5–50/1
	Cs-Fe^103^	15.7	–**/303	10–200/10
	CHMs^104^	10.4	6.5/293	1–250/1
	Cs-Zn^67^	7.4	4.0/293	1–15/0.5
	CHs^96^	6.7	2.5/–**	0.5–25/1
Other adsorbents	ALS-B^105^	46.08	5.3/298	0–70/–**
	MCM-41 silica^106^	21.01	6.0/–**	20–100/2
	Bentonite modified with La(III)^107^	14.0	6.0/298	0.5–80/0.75
	Kaolinite^108^	4.1–5.4	5.0/295	0.6–1860/20
	Cocoa shell biochar^109^	1.48	9.0/–**	0.1–50/–**

*– the best own result for actual phosphate(V) concentration of 9.76 mg/dm^3^, **– no available data in literature.

Significance values are in bold.

### Application of Ce-CTS adsorbent for treating environmental samples

Due to the identified experimentally high affinity of the Ce-CTS adsorbent for phosphates(V) adsorption from the aqueous model aqueous solutions, one of the last stages was to investigate the performance of the obtained adsorbent in real solutions–environmental samples. For this purpose, the phosphates(V) adsorption capabilities from real wastewater and natural surface water samples for the Ce-CTS adsorbent were determined.

In the batch adsorption method, as a result of sewage and surface water treatment using cerium(IV)-modified chitosan-based hydrogels with mass ratio cerium : chitosan 1:4 (20% wt. cerium(IV) dispersed), removal of phosphates(V) was observed. This experimental study examined verified the phosphorus removal capabilities from two representative surface waters: a fresh lake and seawater, and two different wastewaters: typical municipal and typical industrial one. The initial characteristics of these waters and the quantitative effect of their treatment using Ce-CTS adsorbent are presented below in Table 3.

**Table 3:** Characteristics of two surface water and two wastewater samples–before and after adsorption of phosphates(V). Ce-CTS (20% wt. of cerium) hydrogel dose: 20 g/dm^3^ (0.8 g/dm^3^ for dry mass); interfacial contact time: 24 h; temperature: 20 ± 1 °C. The final results as chemical element concentrations (before and after the adsorption) are presented (as the mean of two results and with the calculated standard deviation: $\overline{x } \left(\pm SD\right)$ [mg/dm^3^]).

Name of sample / parameter	Surface water I		Surface water II		Wastewater I		Wastewater II	
	before	after	before	after	before	after	before	after
pH	7.39	7.73	8.71	7.48	7.04	7.74	8.17	8.59
Conductivity [mS/cm]	0.67	0.51	65.60	59.8	0.64	0.54	20.16	18.7
Na	26.352 (± 0.403)	26.251 (± 0.111)	1,583.253 (± 80.564)	1,491.934 (± 19.383)	20.411 (± 0.312)	10.287 (± 0.137)	1,522.727 (± 125.34)	1,198.927 (± 124.503)
K	4.851 (± 0.882)	4.579 (± 0.037)	162.763 (± 13.343)	122.414 (± 11.028)	11.323 (± 0.184)	2.517 (± 0.087)	1,095.225 (± 67.673)	1,079.54 (± 9.975)
Li	–*	–*	–*	–*	–*	–*	0.170 (± 0.024)	0.159 (± 0.005)
Mg	22.972 (± 1.972)	19.798 (± 1.911)	139.652 (± 4.489)	104.169 (± 4.121)	21.069 (± 1.904)	16.423 (± 0.543)	140.870 (± 0.380)	139.262 (± 0.897)
Ca	60.699 (± 0.746)	51.599 (± 0.442)	220.743 (± 2.169)	114.759 (± 4.214)	85.453 (± 2.400)	65.365 (± 8.360)	72.301 (± 5.397)	57.384 (± 3.845)
Mn	0.137 (± 0.008)	0.004 (± 0.001)	0.007 (± 0.001)	0.006 (± 0.001)	0.114 (± 0.011)	0.004 (± 0.001)	0.171 (± 0.028)	0.025 (± 0.002)
Cu	–*	–*	–*	–*	–*	–*	0.018 (± 0.006)	0.017 (± 0.001)
Ni	–*	–*	–*	–*	–*	–*	0.176 (± 0.004)	0.212 (± 0.024)
Co	–*	–*	–*	–*	–*	–*	0.014 (± 0.001)	0.013 (± 0.001)
Fe	0.011 (± 0.001)	0.005 (± 0.001)	0.072 (± 0.011)	0.004 (± 0.001)	0.044 (± 0.002)	0.008 (± 0.001)	2.486 (± 0.343)	1.545 (± 0.221)
Al	0.012 (± 0.003)	0.011 (± 0.009)	0.036 (± 0.010)	0.016 (± 0.004)	0.016 (± 0.006)	0.007 (± 0.001)	0.453 (± 0.064)	0.381 (± 0.007)
B	0.176 (± 0.045)	0.172 (± 0.045)	3.400 (± 0.202)	2.851 (± 0.074)	0.073 (± 0.005)	0.029 (± 0.005)	9.817 (± 0.541)	9.264 (± 0.556)
S	32.843 (± 0.357)	32.194 (± 0.255)	881.554 (± 33.197)	792.545 (± 34.256)	8.975 (± 0.510)	7.620 (± 0.070)	86.468 (± 1.380)	85.291 (± 1.506)
Si	0.992 (± 0.015)	0.430 (± 0.077)	–*	–*	6.378 (± 1.243)	5.790 (± 0.087)	14.617 (± 0.370)	13.870 (± 0.128)
Ce	0.011 (± 0.001)	0.011 (± 0.005)	0.023 (± 0.004)	0.021 (± 0.005)	0.011 (± 0.001)	0.023 (± 0.005)	0.023 (± 0.003)	0.130 (± 0.002)
P-PO_4_	0.4092 (± 0.024)	0.105 (± 0.034)	0.347 (± 0.010)	0.120 (± 0.001)	0.347 (± 0.027)	0.112 (± 0.002)	12.153 (± 0.464)	4.725 (± 0.010)
Phosphates(V) removal [%] Adsorption capacity [mg/g]	74.3 0.380		65.4 0.283		67.9 0.294		61.19.284	

Surface water I: Fresh water collected from the “Pogoria” water reservoir located in the region of Silesia; Surface water II: Seawater collected from the Baltic Sea in the northern part of Poland; Wastewater I: Municipal wastewater collected from a water treatment company located in the “Opole” region in south-western Poland; Wastewater II: Industrial wastewater collected from a company located in the “Wielkopolska” region in the Midwest of Poland; –*: Concentration below the analytical detection limit.

The results confirmed the applicability of the cerium(IV) chitosan-based adsorbent in the removal of orthophosphate(V) ions from the real environmental samples. The orthophosphates(V) removal efficiency is not at the same level as in the case of laboratory-based matrix-free solution. However, the percentage of phosphates(V) removal reaches a relatively high value, about 61.1–74.3% level for the samples studied. The highest phosphates(V) removal efficiency corresponded to surface water I, of less chemically complex matrix than seawater. An analogy was observed for wastewater samples—the adsorption of phosphates(V) was higher for municipal wastewater of simpler composition than in case of industrial wastewater. In the case of an industrial wastewater sample, the identified adsorption capacity (9.3 mg/g) is similar to that for adsorption studies in solution without any matrix—see Fig. 3 (q = 10.4 mg/g and q = 8.3 mg/g for pH = 7 and 8, respectively – while other conditions were: initial concentration = 9.3 mg/dm^3^ P-PO_4_, contact time = 48 h and temperature = 20 °C).

Furthermore, it is significant to discuss also the leaching of cerium filling from the tested adsorbent structure as the effect of contact with more complex real liquid matrices.

From the Table 3 it can be noticed, that there is no concentration increase for cerium after the adsorption of phosphates(V) in both surface water samples (in instrumental analytical tolerance).

The trace concentration of cerium determined in both wastewater samples after the adsorption requires further studies, however the loss of cerium is less than 0.01%.

Considering this, some future technology-oriented research is planned–identification of combinations of chemical components potentially responsible for increased leaching of cerium(IV) from adsorbent structures. In addition to the scientific interpretation of surface phenomena, these studies will provide practical design-oriented information on the initial technological system intended for the initial, preliminary purification of wastewater from these substances harmful to the adsorbent, so that in the next technological stage its separation capabilities can be fully used.

It may be concluded that these specific conditions were observed only in wastewater samples, of more complex chemical composition, which requires more technological research. However, in case of surface waters the new adsorbent proved to be stable (no cerium(IV) leaching effect), thus can be directly used for environmental applications towards surface waters treatment.

In addition to the possible removal of phosphates(V), the resulting adsorbent is capable of removing from multicomponent aqueous solutions also other metals. To a greater extent, the removal (adsorption) effect is observed for such metals as manganese or iron. This increases the potential of new cerium(IV) chitosan-based adsorbent for use in wastewater treatment or in the revitalization of water bodies. The problem here may be the use of this adsorbent for selective adsorption of phosphates(V). For this purpose, it would be necessary to modify the tested adsorbent using, for example, ion imprinting technique.

### Mechanism of adsorption

Considering the interpretation of the FTIR, XPS, SEM and XRD analyses above, and literature data^49,110^, it can be concluded, that during the gelation process of the chitosan beads with cerium(IV) salt additive in NaOH solution, reactive functional groups present in chitosan, -NH_2_ and -OH, having free electron pairs, were coordinating with the Ce^4+^ ions. It resulted in a formation of dispersed cerium dioxide built into the chitosan backbone via the amino and hydroxyl groups.

Modification of chitosan with cerium(IV) results in the enhancement of the properties of chitosan in the increase in pH value of the point of zero charge (pH_PZC_). It was shown that the introduction of Ce^4+^ ions into the chitosan skeleton increased the pH_PZC_ value up to 8.8 (compared to 7.4–7.5 range determined for non-crosslinked CTS^102,111^), thus expanding the range of the adsorption process pH operation and increasing the percentage of phosphorus compounds removal from 24.9 up to 97.5%. The pH_PZC_ is an extremely important parameter in the case of removing phosphates(V) from natural waters. The hydrogel obtained from non-crosslinked chitosan requires acidic pH of the water from which phosphates(V) are adsorbed because the negative charge of the surface of the hydrogel granules occurs at a neutral and alkaline pH and negative phosphate(V) ions are repulsed from the surface of the adsorbent. In natural waters, a neutral or slightly alkaline pH is observed, hence the modification of the hydrogel surface by CeO_2_ eliminates the inconvenience associated with the possible correction of the water pH to the acidic pH range required when using unmodified hydrogel.

**Figure 8:**
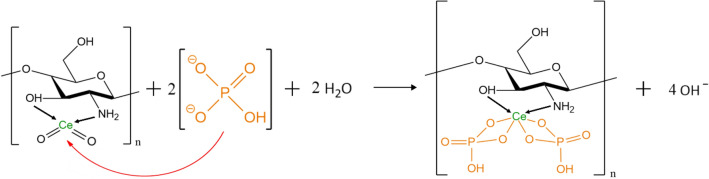
The possible mechanism of phosphate(V) adsorption on Ce-CTS hydrogel beads surface.

The adsorption of phosphate(V) anions on the Ce-CTS adsorbent at acidic, neutral or slightly alkaline pH is presumably the result of electrostatic attraction between the positively charged surface of Ce-CTS hydrogel and the negatively charged phosphate(V) ionic species (H_2_PO_4_^-^ or HPO_4_^2-^ depending on pH). However, the dominant adsorption mechanism is the chemical interaction between the phosphate(V) anions and cerium(IV) resulting in the formation of a stable complex (Fig. 8). In addition to Ce-PO_4_ binding, direct adsorption of phosphates(V) with the participation of free and active chitosan amino groups through the interaction via hydrogen bonding (NH_3_^+^ ---- ^-^OPO(OH)_2_) is also possible^112^, which was confirmed by the FTIR analysis in this study.

## Methods

### Materials and reagents

All the chemical reagents employed in the study were of analytical reagent grade. Sigma-Aldrich potassium dihydrogen phosphate(V) was used to prepare the starting aqueous phosphates(V) solution used for the adsorption process. The water used during the research was purified by a Millipore Elix 10 system (Merck Millipore). Basic standard solution of phosphorus in the form of potassium dihydrogen orthophosphate(V) (1 g/dm^3^ P-PO_4_) and cerium standard solutions of 1 g/dm^3^ in the form of cerium(III) nitrate(V) and other element standard solutions of 1 g/dm^3^ were supplied by Merck. Reagents used for the adsorbent synthesis: chitosan (molecular weight 600,000–800,000 kg/kmol) was purchased from Acros Organics (New Jersey, USA), acetic acid was produced by Avantor Performance Materials Poland S.A. (Gliwice, Poland), sodium hydroxide was supplied by Chempur (Piekary Śląskie, Poland). Nitric acid used for hydrogel dissolution and hydrochloric acid needed to control of solutions pH were purchased from Avantor Performance Materials Poland S.A. (Gliwice, Poland).

## Ce-CTS adsorbent preparation and methods of analyses

**Figure 9:**
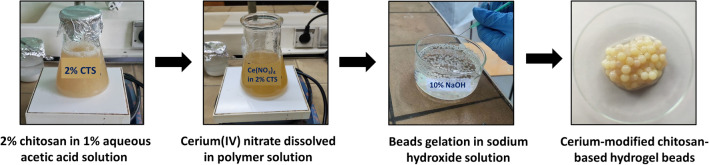
The chitosan-based composite hydrogel – its synthesis scheme.

Synthesis of the chitosan-based composite hydrogel beads was carried out as follows. A 5 g of chitosan polymer was dissolved in 250 cm^3^ of 1% acetic acid (aqueous solution), using a magnetic stirrer. To such prepared solution, cerium(IV) nitrate in the form of hexahydrate was introduced in (cerium: chitosan) mass ratios of 1:5, 1:4, 2:5 2:4, 3:5 and then dispersed using a magnetic stirrer. The mixture thus formed was infused into a 10% sodium hydroxide aqueous solution to crosslink the hydrogel using syringes with a 0.9 mm diameter needle. The resulting adsorbents were subjected to a rinse with deionised water to wash the residual aqueous solution of sodium hydroxide out of the hydrogel structure. Figure 9 shows the chitosan-based composite hydrogel synthesis scheme.

A portion of the obtained hydrogel was analysed by ICP-OES technique to study the composition of the composite samples obtained. The hydrogels were dried and then dissolved in concentrated aqueous solution of nitric acid(V). The resulting mineralised material was diluted accordingly and analysed. The hydrogels were analysed both before and after the phosphates(V) adsorption process. To prepare the adsorbent samples for analysis by FTIR, XRD and XPS techniques, the hydrogels were dried and then pulverised in an agate mortar. XRD and IR analyses were done on adsorbents with a cerium/polymer ratio of 1:4.

FTIR analysis was performed using a Spectrum Two spectrometer (Perkin Elmer, Waltham, MA, USA). Microscopic images of the adsorbents were obtained using a Phenom ProX SEM scanning electron microscope (Phenom-World Bv, Eindhoven, Netherlands). XRD analysis was carried out using a SEIFERT 3003TT diffractometer (Seifert, Ahrensburg, Germany) with Cu X-ray tube (kλ1 = 1.540598 Å, kλ2 = 1.544426 Å and kβ = 1.39225 Å). The powder sample was analysed between 10° and 80° of 2Theta using a 0.05° step. XPS investigations were done in UHV multi-chamber experimental setup with PREVAC EA15 hemispherical electron energy analyzer fitted with 2D-MCP detector (Prevac sp. z o.o., Rogów, Poland). The system base pressure was equal to 9·10^–9^ Pa. The X-ray source (PREVAC dual-anode XR-40B, Al-Kα excitation line with energy 1486.60 eV) was utilized for sample excitation. Pass energy (PE) was set to 200 eV for survey spectra (scanning step of 0.8 eV) and to 100 eV for particular high-resolution energy regions (scanning step of 0.05 eV). All measurements were done with a normal take-off angle and the curved analyzer exit slit (0.8 × 25 mm) for the highest energy resolution and limitations of the possible diffraction effects. The binding energy (BE) scale of the analyzer was referred to Au 4f_7/2_ (84.0 eV) region of the gold-covered sample placed at the same sample stage^113^. The acquired spectra were processed and using CASA XPS® software (version 2.3.23PR01). The components were fitted with sum of Gauss (30%) and Lorenz (70%) functions and with use of Shirley function for the background subtraction.

The procedure for the determination of the point of zero charge (pH_pzc_) of the Ce-CTS adsorbent is given in Supplementary Information.

### Batch adsorption experiments

#### Determination of adsorption capacity

Determination of the adsorption capacity was carried out for model aqueous phosphate(V) ions solutions, with a phosphates(V) concentration of 0.3 mM and at pH 7 (except for tests for different pH values from within the 4–10 range). The solutions were prepared by dissolving an appropriate mass of potassium dihydrogen phosphate(V) in deionised water in a volumetric flask. The appropriate pH was obtained by dropping a corresponding volume of aqueous solution hydrochloric acid into the resulting solution. Hydrogel doses—each time 1 g, dried with blotting paper—were introduced into conical flasks with a ground glass stopper. The adsorbents prepared this way were filled with 50 cm^3^ of phosphates(V) solution and placed on a shaker (parameters: 48 h, 180 rpm, T = 20 °C). After shaking, the spent adsorbent was separated from the solution by filtration on analytical filters (FILTRAK, hard filters, type 390, 84 g/m^2^). The filtrate obtained was analysed employing the inductively coupled plasma atomic emission spectroscopy (ICP-OES). The reference solution was represented by the solution with which the hydrogels were filled. Each trial was performed at least twice.

#### Determination of adsorption process kinetics

The adsorption kinetics in the investigated process system was determined using 10 g of chitosan-based composite hydrogel with mass ratio cerium : chitosan 1:4 (20% wt. cerium(IV) dispersed). The adsorbent prepared this way was poured into 500 cm^3^ of a phosphates(V) aqueous solution with a concentration of 0.3 mM and for pH 7. The prepared system was set to shake. Using an automatic pipette, 1 cm^3^ of the supernatant solution was taken, poured into 5 cm^3^ flasks and then filled up to the mark with deionised water. Samples were taken: 5, 10, 20, 40, 60, 120, 180, 240, 420, 1440 (24 h), 1800 (30 h) and 2880 (48 h) minutes after the start of shaking (180 rpm, room temperature T = 20 °C). The samples collected were analysed by ICP-OES technique. The stock solution was considered as the reference solution.

#### Determination of the ratio of solution volume to adsorbent mass

Determination of the solution volume to adsorbent mass ratio was done by successively adding adsorbent mass of: 0.05; 0.075; 0.10; 0.25; 0.50; 0.75; 1.00; 2.50 g into 100 cm^3^ ground-glass conical flasks and then pouring 50 cm^3^ of a 0.3 mM phosphates(V) solution. The prepared suspensions were shaking for 48 h. Then the supernatant solutions were separated from the adsorbent by filtration. The filtrates were analysed by ICP-OES.

Independent research was carried out to determine the most favourable chemical structure of the adsorbent composite, i.e. the polymer : cerium ratio. The study included six types of adsorbents: hydrogel without any dispersed additive (non-cross-linked CTS) and composite hydrogels with the mass ratio of cerium to chitosan set as: 1:5, 1:4, 2:5, 2:4 and 3:5, respectively.

#### Determination of the adsorption process isotherms

Determination of the adsorption process isotherms was carried out in a similar way as for the adsorption capacity, except for the solutions with which the hydrogels were contacted represented different concentrations: 0.1; 0.5; 1; 2; 5; 10; 15; 20; 30; 50; 75; 100; 500 mg/dm^3^. These were also regarded to be reference solutions. All adsorption isotherms were determined for a chitosan-based composite adsorbent—hydrogel with 20% wt. cerium(IV) dispersed (mass ratio cerium : chitosan 1:4) considering the best adsorption performance identified during the preliminary adsorption capacity tests.

#### Determination of the temperature effect on the adsorption process

Determination of the effect of temperature on the adsorption process was carried out using 1 g of hydrogel samples into 100 cm^3^ conical flasks closed to the ground and then pouring a 0.3 mM of phosphates(V) solution. The flasks were closed with ground glass stoppers and then placed on a thermostatic shaker for 24 h. Three measurements were carried out for temperatures of: 298, 308 and 318 K. Immediately after shaking, the solutions were quickly filtered and the resulting filtrates were analysed.

#### Determination of adsorption process on environmental samples

Determination of the adsorption efficiency was carried out for actual aqueous environmental samples: two surface water samples and two wastewater ones. Firstly, the samples were filtered by analytical filters (FILTRAK, hard filters, type 390, 84 g/m^2^) to separate solids. Hydrogel doses of 1 g, dried with blotting paper—were introduced into conical flasks with a ground glass stopper. The adsorbents prepared this way were filled with 50 cm^3^ of actual samples and placed on a shaker (parameters: 48 h, 180 rpm, T = 20 °C). After shaking, the spent adsorbent was separated from the solution by filtration on analytical filters (FILTRAK, hard filters, type 390, 84 g/m^2^). The filtrate obtained was analysed employing the inductively coupled plasma atomic emission spectroscopy (ICP-OES). The reference solution was represented by the solution with which the hydrogels were filled. Each test was performed at least twice.

#### ICP-OES method

The determination of phosphorus and cerium concentrations in the analysed solutions was carried out by inductively coupled plasma optical emission spectroscopy (ICP-OES).

Analyses were performed on analytical lines for phosphorus λ = 213.618 nm; λ = 214.914 nm and for cerium λ = 407.347 nm; λ = 418.659 nm; λ = 446.021 nm. The range of the calibration curve for phosphorus was 0.1–100 mg/dm^3^, whereas for cerium 0.05–20 mg/dm^3^. The spectrometer used was a Varian 710-ES (Varian, Mulgrave, Victoria, Australia).

Multi-element analysis for the adsorption on actual samples was performed on analytical lines for sodium λ = 330.237 nm; λ = 568.821 nm, potassium λ = 766.491 nm; λ = 769.897 nm, lithium λ = 460.289 nm; λ = 610.365 nm, magnesium λ = 279.078 nm; λ = 285.213 nm, calcium λ = 317.933 nm; λ = 422.673 nm, manganese λ = 257.610 nm; λ = 260.568 nm, copper λ = 324.754 nm; λ = 327.395 nm, nickel λ = 216.555 nm; λ = 230.299 nm, cobalt λ = 228.615 nm; λ = 238.345 nm, iron λ = 238.204 nm; λ = 259.940 nm, aluminum λ = 308.215 nm; λ = 394.401 nm, boron λ = 182.577 nm; λ = 249.678 nm, sulphur λ = 180.669 nm; λ = 181.972 nm, silica λ = 252.851 nm; λ = 288.158 nm. The range of the calibration curve for these elements was various and ranged between 0.001–100 mg/dm^3^.

## Conclusions

The work presents an innovative technological method of phosphorus compounds removal from aqueous systems based on its adsorption on chitosan-based hydrogel with a dispersed cerium(IV) oxide (Ce-CTS). Modification of non-crosslinked chitosan with cerium(IV) results in the enhancement of the properties of chitosan in the range of physical adsorption mode (increase in pH value of point of zero charge), as well as chemical adsorption (through the presence of Ce(IV) that demonstrates chemical affinity to phosphate(V) anions).

From the results it can be clearly seen, that the cerium(IV)-modified chitosan-based hydrogel maintains a high phosphates(V) removal efficiency and rate regardless of the solution pH in the 4–7 range, most favourable at pH 4. The percentage of phosphates(V) removal equals to 97.5%, is. 4-times higher than for the CTS without any dispersed additive. When the Ce-CTS adsorbent was used to purify the solution from phosphates(V) with an excess concentration of approx. 0.3 mM (9.76 mg/dm^3^), the highest adsorption capacity of 71.7 mg/g was reported, under the following process conditions: pH = 7, an adsorbent dose = 1 g/dm^3^, temperature = 293 K. Comparing this value with the maximum adsorption capacities of other alternative adsorbents capable of removing phosphates(V) and provided in the accessible literature, it turns out, that this value is relatively high (see Table 2). Furthermore, phosphates(V) adsorption studies with the Ce-CTS adsorbent using natural surface waters showed only a slight decrease in the efficiency of phosphates(V) removal compared to very simply model solutions containing no other components, which are however typical for these surface waters, which mean confirms a great and reliable technological potential of the proposed adsorbent for the use in water treatment.

Moreover, Ce-CTS adsorbent demonstrates higher mechanical strength and better resistance to changeable chemical conditions than the CTS alone and there is no need to cross-link it anymore. The best agreement between the measurement data and the modelling results was obtained for the Hill isotherm model, which forecasts the maximum adsorption capacity of ca. 52.7 mg/g on the basis of the available equilibrium data course. However, for low concentrations of phosphates(V) in initial solution, the experimental data expressed a good compatibility with Langmuir model of adsorption isotherm and—for moderate concentrations—Freundlich adsorption isotherm gave a better representation of the experimental data course. Thermodynamic study showed that the process is exothermic and the adsorption proceeds spontaneously. From the kinetic point of view, pseudo-second-order model explained the phosphates(V) adsorption data for Ce-CTS adsorbent. Independent application of Weber-Morris kinetic model identified clearly the intraparticle diffusion mechanism, however only as one of the potential mass transfer mechanisms involved.

The excellent phosphates(V) adsorption properties of the presented Ce-CTS adsorbent are due to two properties of chitosan: the ability to modify its structure with a dispersed cerium(IV) oxide, which demonstrates relatively high specific surface area and high affinity for phosphates(V), and the ability to form a hydrogel, which improves phosphates(V) penetration into the inner adsorbent structure and facilitates solid phase separation from the liquid phase after the adsorption process is completed what is advantageous for technological applications.

In further research, phosphates(V) desorption tests to reuse hydrogels to phosphates(V) removal (cyclic use) are planned to be carried out. The presented study on the effect of pH on phosphates(V) adsorption indicates the possibility of desorption of phosphates(V) in alkaline solutions. This conclusion has already been confirmed in the conducted preliminary desorption studies.

Future research work of the team will focus on identifying the factors responsible for further increasing the adsorption capacity of the tested adsorbent.

Due to the planned application in environmental protection domain, in particular for the prevention of eutrophication phenomena of natural water reservoirs, the future research work will focus on the study of the possibility of simultaneous co-adsorption of phosphorus and nitrogen compounds, with the systematic optimisation of the tested new adsorbent structures.

An important problem for future experimental work will also be the study of the impact of various different compounds co-present in natural aquatic ecosystems, conditioning, through various inhibition or catalytic processes, potentially different adsorption capabilities of the tested system depending on the complex process environment.

Identification of the most harmful compounds (and their concentrations) influencing the efficiency of the complex adsorption process of phosphorus compounds (and possibly nitrogen compounds) will also allow for rational design of e.g., the alternative initial water pre-purification process of these substances, which will result in a more effective use of the adsorption capacity of the described class of adsorbents in the consecutive stage of the technological process.

### Supplementary Information

Supplementary Information.

## Data Availability

The datasets used and/or analysed during the current study are available from the corresponding author (J.K.) on reasonable request.

## Abbreviations

CTS: Chitosan without any modification
Ce-CTS: Chitosan modified with Ce(IV)
P-Ce-CTS: Ce-CTS after phosphates(V) adsorption
FTIR: Fourier-Transform Infrared Spectroscopy
ICP-OES: Inductively coupled plasma atomic emission spectroscopy
SEM–EDS: Scanning Electron Microscopy with Energy Dispersive XRay Spectroscopy
XRD: X-Ray Diffraction
XPS: X-Ray Photoelectron Spectroscopy
x mg/dm^3^ P-PO_4_: Concentration of phosphorus calculated on phosphates(V) basis
pH_PZC_: pH value of the point of zero charge of adsorbent
