# Supplementary material for: Cerium(IV) chitosan-based hydrogel composite for efficient adsorptive removal of phosphates(V) from aqueous solutions
Source: Sci Rep. 2023 Aug 11;13:13049. doi: 10.1038/s41598-023-40064-1 (PMC10421956; doi:10.1038/s41598-023-40064-1)
Supplement: Supplementary file 1 — Supplementary Information. [file 41598_2023_40064_MOESM1_ESM.pdf]

Cerium(IV) chitosan-based hydrogel composite for efficient adsorptive removal of phosphates(V) from aqueous solutions

Łukasz Wujcicki, Tomasz Mańdok, Wiktoria Budzińska-Lipka, Karolina Pawlusińska, Natalia Szozda, Gabriela Dudek, Krzysztof Piotrowski, Roman Turczyn, Maciej Krzywiecki, Alicja Kazek-Kęsik & Joanna Kluczka*

*email: joanna.kluczka@polsl.pl

Supplementary Information

SEM-EDS

Table S1. The average diameter of the pore size in chitosan-based hydrogel, modified with cerium(IV) - before and after phosphates(V) adsorption.

Hydrogel type	Average pore diameter, µm
CTS	6.69 ± 0.43
Ce-CTS	6.36 ± 0.67
P-Ce-CTS	6.97 ± 0.41

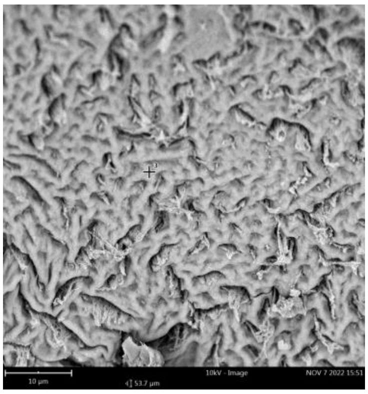

Figure S1. The wrinkled maze-like structures of the freeze-dried bead's surface of the chitosan-based hydrogel.

XRD

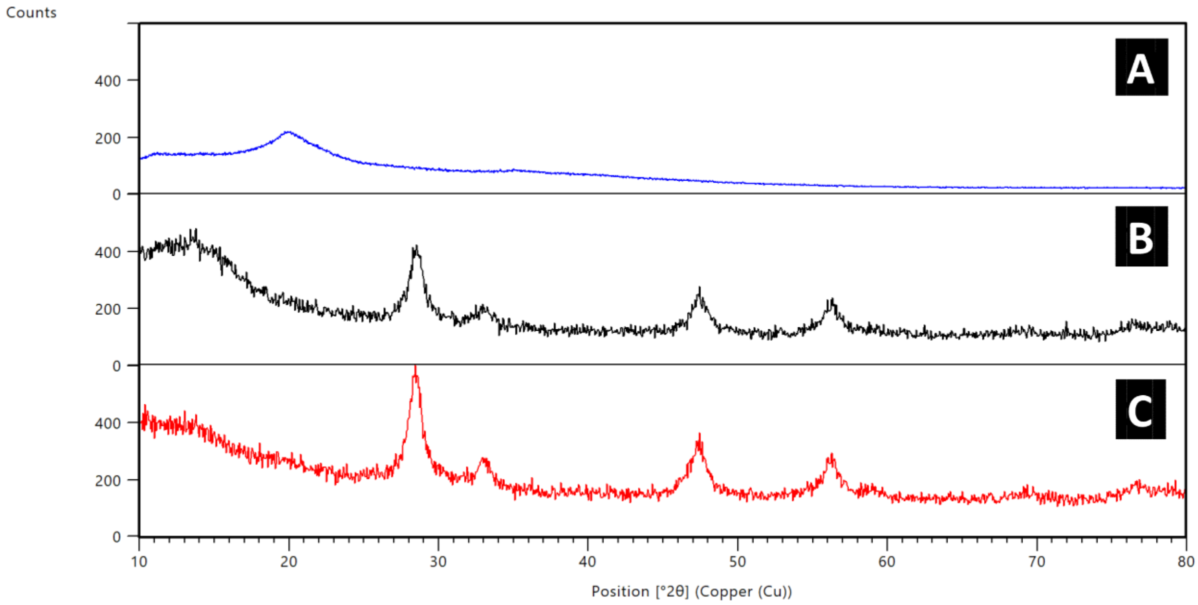

Fig. S2 The XRD spectra of the chitosan (**A** - blue), chitosan modified with cerium(IV) before (**B** - black) and after phosphates(V) adsorption (**C** - red)

Phosphate(V) adsorption

Adsorption capacity of the cerium-modified chitosan-based adsorbent in respect to phosphates(V), as well as the percentage of phosphates(V) removal, were calculated based on the (S1) and (S2):

$$q_t = \frac{(C_0 - C_t) \cdot V}{m_s} \quad \text{(S1)}$$

$$R\% = \frac{(C_0 - C_t)}{C_0} \cdot 100\% \quad \text{(S2)}$$

In equations (S1) and (S2) parameter q_t [mg/g] is the equilibrium mass of phosphorus, corresponding to the given time of the process *t*, chemically as phosphates(V), adsorbed on a unit mass of adsorbent, R% is the percentage of phosphorus removal from solution, C_0 and C_t [mg/dm^3] are the initial and corresponding to the given time of the process *t* concentration of phosphorus in the chemical form of phosphates(V) in solution, respectively, V [dm^3] is the volume of phosphates(V) aqueous solution, while m_s [g] is the mass of dry adsorbent used in the system.

Procedure of the point of zero charge (pH_{pzc}) determination for the Ce-CTS adsorbent was the following [S1 [111]]. To a series of polyethylene bottles containing 40 cm^3 of 0.01 M KNO_3 solution each, different volumes of either 0.1 M HCl or 0.1 M NaOH solution, appropriately, were added to obtain pH values from 4 to 10. The total volume of solution in each bottle was 50 cm^3 by adequate addition of demineralized water (Millipore Elix 10 system (Merck Millipore)). After 2 h of equilibration, the pH values (pH_i) were identified and 1 g of the hydrogel beads to each solution was added. After 72 h of equilibration with discontinuous (in cycles 15 min. per h) shaking, the pH value of the supernatant liquid in each bottle was identified (pH_f). The difference between pH_i and pH_f (as ΔpH) was then plotted against each tested pH_i. The solution's pH at which ΔpH = 0 means "the pH_{pzc}" of the sample.

Kinetics [72,73]

Pseudo-first-order kinetic model (S3):

$$\log(q_e - q_t) = \log q_e - \frac{k_1}{2.303}t \qquad (S3)$$

Pseudo-second-order kinetic model (S4):

$$\frac{t}{q_t} = \frac{1}{k_2 q_e^2} + \frac{1}{q_e}t \qquad (S4)$$

where: t is the time of an adsorption process (representing interfacial contact time), k_1 and k_2 are the rate constants for the pseudo-primary and pseudo-secondary kinetic models, respectively, q_e is the adsorption capacity at equilibrium, and q_t is the adsorption capacity effect for a given interphase contact time (usually termed: practical equilibrium capacity).

Intraparticle diffusion model by Weber and Morris (S5) [78,79,80,81,82]:

$$q_t = K_{i\,diff} t^{0.5} + C \qquad (S5)$$

where: q_t is the adsorption capacity of phosphorus for time t [mg/g], t is an adsorption process time (interfacial contact) [min], $K_{i\ diff}$ is a rate constant for intraparticle diffusion [mg/(g · min$^{0.5}$)], and C is a constant of the Weber and Morris model [mg/g].

Based on the value of the rate constant for intraparticle diffusion $K_{i\ diff}$, the value of D – intraparticle diffusion coefficient – can be determined from the formula (S6):

$$K_{i\,diff} = \frac{6 \cdot q_e}{r \cdot (\frac{D}{\pi})^{0.5}} K_{i\,diff} t^{0.5} \qquad (S6)$$

where [83]: r –is a radius of adsorbent particle [cm], D is an intraparticle diffusion coefficient [cm^2/min], and q_e is the adsorption capacity at equilibrium [mg/g].

Table S2. Kinetic parameter values for phosphates(V) adsorption on the cerium-modified chitosan-based hydrogel at temperature 20±1°C, experimentally determined composite adsorbent capacity was 9.61 mg/g.

Pseudo-first-order kinetic model		
q_1 (mg/g)	k_1 (g/(mg · h))	R^2
5.98	0.0012	0.9224
Pseudo-second-order kinetic model		
q_2 (mg/g)	k_2 (g/(mg · h))	R^2
9.75	0.0012	0.9985
Formal Weber-Morris model (similar to [77,85])		

C_1 (mg/g)	$K_{i\ diff\ (1)}$ (g/(mg · min$^{0.5}$))	R^2
0.43003	0.43973	0.915179
Weber-Morris model, intraparticle diffusion		
C_2 (mg/g)	$K_{i\ diff\ (2)}$ (g/(mg · min$^{0.5}$))	R^2
5.74992	0.07703	0.966132

Isotherms [91-94]

Langmuir model

$$q_e = \frac{q_m K_L C_e}{1+K_L C_e} \tag{S7}$$

where:
q_e – equilibrium surface concentration of the adsorbate (mass ratio) on adsorbent, mg/g
q_m – maximum surface concentration of the adsorbate (single-layer adsorption) (mass ratio) on adsorbent, mg/g
K_L – Langmuir isotherm constant, dm^3/mg
C_e – equilibrium concentration of adsorbate in the solution, mg/dm^3

$$R_L = \frac{1}{1+K_L C_0} \tag{S8}$$

where:
R_L – separation factor (equilibrium parameter)
C_0 – the highest initial concentration of adsorbate in the solution, mg/dm^3

Freundlich model

$$q_e = K_F {C_e}^{1/n} \tag{S9}$$

where:
K_F – Freundlich isotherm constant, (dm^3/mg)$^{1/n}$
$1/n$ – exponent of the Freundlich isotherm model (representing adsorption intensity or adsorbent surface heterogeneity)

Redlich-Peterson model

$$q_e = \frac{K_{RP} C_e}{1+a_{RP} {C_e}^g} \tag{S10}$$

where:
K_{RP} –Redlich-Peterson isotherm constant, (dm^3/mg)(mg/g) = (dm^3/g)
a_{RP} – Redlich-Peterson model parameter, (dm^3/mg)^g
g – Redlich-Peterson model exponent

Jovanović model

$$q_e = q_J\left[1 - exp\left(-K_J C_e\right)\right] \tag{S11}$$

where:
q_J - maximum surface concentration of the adsorbate (single-layer adsorption) (mass ratio) on the adsorbent, mg/g
K_J - Jovanović isotherm constant, dm^3/mg

Hill model

$$q_e = \frac{q_H C_e^{n_H}}{K_H + C_e^{n_H}} \qquad \text{(S12)}$$

where:

q_H - maximum surface concentration of the adsorbate (mass ratio) on adsorbent, $(mg/g)(dm^3/mg)^{nH}$

n_H – exponent of the Hill model theoretically associated with binding interaction

K_H –Hill model constant, $(mg/dm^3)^{nH}$

Table S3. Adsorption isotherm models with their parameter values identified experimentally for phosphates(V) adsorption on the cerium-modified chitosan-based hydrogel adsorbent (20 ± 1°C).

No	Model	Equation	Parameters
1	Langmuir	$q_e = \frac{q_m K_L C_e}{1 + K_L C_e}$	q_m = 38.553 mg/g<br>K_L = 0.075 dm^3/mg<br>R^2 = 0.943
2	Freundlich	$q_e = K_F C_e^{1/n}$	K_F = 8.832 $(dm^3/mg)^{1/n}$<br>$1/n$ = 0.261<br>R^2 = 0.951
3	Redlich-Peterson	$q_e = \frac{K_{RP} C_e}{1 + a_{RP} C_e^{g}}$	K_{RP} = 20.957 $(dm^3/mg)(mg/g)$ = (dm^3/g)<br>a_{RP} = 1.711 $(dm^3/mg)^g$<br>g = 0.799<br>R^2=0.981
4	Jovanović	$q_e = q_J[1 - exp(-K_J C_e)]$	q_J = 35.594 mg/g<br>K_J = 0.053 dm^3/mg<br>R^2 = 0.901
5	Hill	$q_e = \frac{q_H C_e^{n_H}}{K_H + C_e^{n_H}}$	q_H = 52.666 $(mg/g)(dm^3/mg)^{nH}$<br>n_H = 0.511<br>K_H = 6.783 $(mg/dm^3)^{nH}$<br>R^2 = 0.981

Thermodynamics

The following equations were considered: (S13) & (S14) [96]:

$$\ln K_c = \frac{\Delta S^{\ominus}}{R} - \frac{\Delta H^{\ominus}}{RT} \qquad \text{(S13)}$$

$$\Delta G^{\ominus} = \Delta H^{\ominus} - T\Delta S^{\ominus} \qquad \text{(S14)}$$

In both equations, $\Delta G^{\ominus}$ is the Gibbs free energy of adsorption, $\Delta H^{\ominus}$ is the enthalpy of adsorption, $\Delta S^{\ominus}$ is the entropy of a thermodynamic system under study, K_c is the adsorption equilibrium constant, and R is the universal gas constant (8.314 J/(mol · K)).

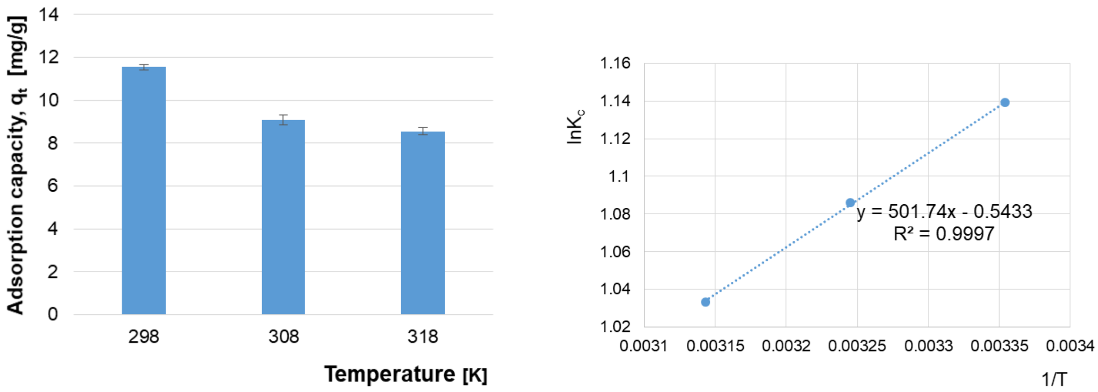

Figure S3. Effect of process temperature on phosphates(V) adsorption on cerium(IV)-modified chitosan-based hydrogel (left panel) and Arrhenius-type correlation between 1/T and the adsorption equilibrium constant lnK_c (right panel). Ce-CTS (20% wt. of cerium(IV)) hydrogel dose: 20 g/dm^3; initial concentration of P-PO_4: 9.3 ± 0.1 mg/dm^3; pH: 7; contact time: 24 h; tested temperature: 298, 308 and 318 K.

Table S4. Thermodynamic parameter values corresponding to different adsorption process temperature values (298 – 318 K)

ΔH° [kJ/mol]	ΔS° [kJ/(mol · K)]	ΔG° [kJ/mol]		
		Temperature [K]		
		298	308	318
-4.20	-4.83 · 10^{-3}	-2.76	-2.71	-2.66
